# Lineage Plasticity in SCLC Generates Non-Neuroendocrine Cells Primed for Vasculogenic Mimicry

**DOI:** 10.1016/j.jtho.2023.07.012

**Published:** 2023-07-16

**Authors:** Sarah M. Pearsall, Stuart C. Williamson, Sam Humphrey, Ellyn Hughes, Derrick Morgan, Fernando J. García Marqués, Griselda Awanis, Rebecca Carroll, Laura Burks, Yan Ting Shue, Abel Bermudez, Kristopher K. Frese, Melanie Galvin, Mathew Carter, Lynsey Priest, Alastair Kerr, Cong Zhou, Trudy G. Oliver, Jonathan D. Humphries, Martin J. Humphries, Fiona Blackhall, Ian G. Cannell, Sharon J. Pitteri, Gregory J. Hannon, Julien Sage, Caroline Dive, Kathryn L. Simpson

**Affiliations:** a Cancer Research UK Cancer Biomarker Centre, University of Manchester, United Kingdom; b Cancer Research UK Manchester Institute, University of Manchester, United Kingdom; c Cancer Research UK Lung Cancer Centre of Excellence, Manchester, United Kingdom; d Department of Radiology, Canary Center at Stanford for Cancer Early Detection, Stanford, California; e Department of Pediatrics, Stanford University, Stanford, California; f Department of Genetics, Stanford University, Stanford, California; g Department of Pharmacology and Cancer Biology, Duke University, Durham, North Carolina; h Faculty of Biology Medicine and Health, Wellcome Centre for Cell-Matrix Research, University of Manchester, United Kingdom; i Department of Life Sciences, Manchester Metropolitan University, Manchester, United Kingdom; j Division of Cancer Sciences, Faculty of Biology, Medicine, and Health, University of Manchester, Manchester, United Kingdom; k Medical Oncology, Christie Hospital National Health Service (NHS) Foundation Trust, Manchester, United Kingdom; l Cancer Research UK Cambridge Institute, University of Cambridge, Li Ka Shing Centre, Robinson Way, Cambridge, United Kingdom

**Keywords:** SCLC, Vasculogenic mimicry, Neuroendocrine tumor, Intratumoral heterogeneity, Tumor plasticity

## Abstract

**Introduction:**

Vasculogenic mimicry (VM), the process of tumor cell transdifferentiation to endow endothelial-like characteristics supporting de novo vessel formation, is associated with poor prognosis in several tumor types, including SCLC. In genetically engineered mouse models (GEMMs) of SCLC, NOTCH, and MYC co-operate to drive a neuroendocrine (NE) to non-NE phenotypic switch, and co-operation between NE and non-NE cells is required for metastasis. Here, we define the phenotype of VM-competent cells and molecular mechanisms underpinning SCLC VM using circulating tumor cell–derived explant (CDX) models and GEMMs.

**Methods:**

We analyzed perfusion within VM vessels and their association with NE and non-NE phenotypes using multiplex immunohistochemistry in CDX, GEMMs, and patient biopsies. We evaluated their three-dimensional structure and defined collagen-integrin interactions.

**Results:**

We found that VM vessels are present in 23/25 CDX models, 2 GEMMs, and in 20 patient biopsies of SCLC. Perfused VM vessels support tumor growth and only NOTCH-active non-NE cells are VM-competent *in vivo* and *ex vivo*, expressing pseudohypoxia, blood vessel development, and extracellular matrix organization signatures. On Matrigel, VM-primed non-NE cells remodel extracellular matrix into hollow tubules in an integrin *β*1–dependent process.

**Conclusions:**

We identified VM as an exemplar of functional heterogeneity and plasticity in SCLC and these findings take considerable steps toward understanding the molecular events that enable VM. These results support therapeutic co-targeting of both NE and non-NE cells to curtail SCLC progression and to improve the outcomes of patients with SCLC in the future.

## Introduction

Patients with SCLC typically present with high circulating tumor cell (CTC) burden and early wide-spread metastasis with a 5-year survival of less than 7%.^1,2^ Despite inter- and intratumoral heterogeneity, SCLC treatment is homogeneous (platinum-etoposide chemotherapy) and responses are short-lived.^2^ Immunotherapy was recently incorporated into the standard of care, albeit benefiting only approximately 15% of people within an unselected subpopulation.^3-5^ As research biopsies present a significant challenge, we pioneered the generation of CTC-derived explants (CDX) from peripheral blood.^6^ CDX faithfully recapitulate the histopathology, recently defined molecular subtypes,^7^ and chemotherapy responses of donor patient tumors.^8^ In SCLC genetically engineered mouse models (GEMMs), NOTCH and MYC co-operate to drive phenotype switching from NE to non-NE cells^9-11^ in which non-NE cells are less tumorigenic but support NE cell expansion *in vivo*,^12^ and in which paracrine signaling between NE and non-NE cells facilitates metastasis.^13^ Functional plasticity accompanied by increased intratumoral heterogeneity and epithelial-to-mesenchymal transition with loss of NE phenotype is observed during chemotherapy resistance.^14^ Induction of the newly described inflamed SCLC subtype, SCLC-I, after chemotherapy also reflects SCLC plasticity and SCLC-I predicts preferential response to immune checkpoint inhibitor combination therapy.^15^ Phenotypic plasticity may explain the almost inevitable relapse and early metastatic spread as tumor cells adopt a variety of behaviors to adapt and thrive in diverse microenvironments,^16^ and thus, strategies to combat plasticity may be essential for effective treatment of patients with SCLC. Although the importance of non-NE cells is recognized, their functions within SCLC tumors are not well understood.

VM is associated with hypoxia, cellular plasticity, and metastasis in several cancer types.^17-20^ We previously reported that VM occurs in SCLC, is associated with worse patient prognosis, and was associated with chemoresistance and faster growth in a xenograft model.^21^ Here, in 25 CDX and 2 GEMMs^22,23^ we found that non-NE cells are pseudohypoxic and transcriptionally primed for VM. We found that perfusable VM vessels are formed by non-NE cells and that NE to non-NE transition is driven by NOTCH in CDX *ex vivo* cultures. In non-NE cells on Matrigel, proteins involved in cell-cell, and cell-extracellular matrix (ECM) adhesion enable collagen remodeling to form hollow tubular networks, a process requiring integrin *β*1. These data suggest that NE and non-NE cells must be targeted to combat VM-supported tumor growth and metastasis.

## Materials and Methods

### Data Code and Availability

Bulk RNA-seq data have been deposited at GEO under the accession number GEO: GSE240789. R scripts used to process RNAseq data are available on GitHub (https://gitlab.com/cruk-mi/cdx-ngs-analysis/). Source data are available from the corresponding author on reasonable request. No algorithms or software were developed in this study. Software that was used is free and open source and details on acquiring them can be found in the associated references.

### Patient Samples

The patients described in this study had samples obtained between February 2012 and December 2017 after informed consent and according to ethically approved protocols as follows: (1) the European Union Molecular mechanisms underlying chemotherapy resistance, therapeutic escape, efficacy, and toxicity (Chemo-RES) study FP6 contract number LSHC-CT-2007-037665 (North West - Greater Manchester West Research Ethics Committee 07/H1014/96); (2) the Tumor Characterization To Guide Experimental Targeted Therapy (TARGET) study (approved by the North-West (Preston) National Research Ethics Service in February 2015, reference 15/NW/0078). Patient metadata can be found in the article by Simpson et al.^8^

### CDX Generation

CDX models were generated as previously described.^8^ In brief, 10 mL of ethylenediaminetetraacetic acid peripheral blood was collected from patients with SCLC enrolled in the ChemoRES study (07/H1014/96). CTCs were enriched by means of RosetteSep (#15167, Stem Cell Technologies, Vancouver, Canada) and subcutaneously implanted into the flank of 8 to 16-week-old non-obese, diabetic, severe combined immunodeficient, interleukin-2 receptor γ–deficient (NSG) mice (Charles River Laboratories International, Inc., Wilmington, MA). CDX models were generated from the patients’ CTCs enriched from blood samples at pre-chemotherapy baseline or at post-treatment disease progression time points (designated P, or PP).^8^

### Disaggregation and Culture of CDX

CDX tumors were grown to approximately 800 mm^3^ and the mice were killed by schedule 1 method. The tumors were removed and dissociated into single cells using the Miltenyi Biotec tumor dissociation kit (#130-095-929 [Miltenyi Biotec, Germany]) following the manufacturer’s instructions on a gentleMACS octo dissociator (#130-096-427 [Miltenyi Biotec]), as previously described.^8^ Single cells were incubated with anti-mouse anti-MHC1 antibody (eBioscience clone, 34-1-2s [ThermoFisher Scientific, Waltham, MA), anti-mouse anti–immunoglobulin G (IgG) 2a+b microbeads and dead cell removal microbead set (Miltenyi Biotec #130-090-101) and applied to an LS column in a MidiMACS Separator (Miltenyi Biotec) for immunomagnetic depletion of mouse cells and dead cells. CDX *ex vivo* cultures were maintained in Roswell Park Memorial Institute (RPMI) 1640 medium supplemented with the following components: 10 nM hydrocortisone, 0.005 mg/mL Insulin, 0.01 mg/mL transferrin, 10 nM *β*-estradiol, and 30 nM sodium selenite; 5 *μ*M Rho kinase inhibitor added fresh (Selleckchem, Y27632 [Houston, TX]), and 2.5% fetal bovine serum added after 1 week at 37°C and 5% carbon dioxide.

### Mouse SCLC Models

RBL2 SCLC (*Trp53^fl/fl^*/*Rb1^fl/fl^*/*Rbl2^fl/fl^*) *Hes1*-green fluorescent protein (GFP) reporter mouse model was generated by the Sage laboratory^22^ and independent cell lines were obtained from the Sage laboratory and designated the references YT326, YT330, and LJS1157. NE (HES1^−^/GFP^−^) and non-NE (HES1^+^/GFP^+^) cells were separated by flow cytometry on the basis of *Hes1*-GFP reporter expression. RPM SCLC (*Trp53^fl/fl^*/*Rb1^fl/fl^*/*Myc^LSL/LSL^*) mice were generated by the Oliver laboratory,^23^ formalin-fixed paraffin-embedded (FFPE) tissue was obtained from the Oliver laboratory (Duke University, Durham, NC) and the mouse model was obtained from The Jackson Laboratory (Bar Harbor, ME) (stock number #029971).

### Mouse Lung Tumor Initiation

For *in vivo* studies with the RBL2 model, tumors were induced in 8- to 12-week-old mice by intratracheal instillation with 4 × 10^7^ plaque-forming units of Adeno-CMV-Cre (Baylor College of Medicine, Houston, TX). For *in vivo* studies with the RPM model, tumors were induced in 6- to 8-week-old mice by nasal inhalation with 10^6^ to 10^8^ plaque-forming units of Adeno-CGRP-Cre (Viral vector core, University of Iowa, Iowa City, Iowa). Viruses were administered in a biosafety level 2 room according to institutional biosafety committee guidelines. Both male and female mice were equally divided between treatment groups for all experiments. To generate RPM cell lines, 7-week-old RPM mice were killed by a schedule 1 method and the lungs were disaggregated with Liberase at 37°C (Millipore Sigma, #5401127001 [Sigma-Aldrich, St. Louis, MO]), according to the manufacturer’s instructions. Cell lines were maintained in RPMI 10% fetal bovine serum.

### Ethics Statement

For *in vivo* studies with the CDX models, all procedures were carried out in accordance with Home Office Regulations (United Kingdom), the U.K. Coordinating Committee on Cancer Research guidelines, and by approved protocols (Home Office Project license 40-3306/70-8252/P3ED48266 and Cancer Research U.K. Manchester Institute Animal Welfare and Ethical Review Advisory Body). For *in vivo* studies with the RBL2 model, mice were maintained according to practices prescribed by the National Institutes of Health at Stanford’s Research Animal Facility (protocol #13565). Additional accreditation of Stanford animal research facilities was provided by the Association for Assessment and Accreditation of Laboratory Animal Care. For *in vivo* studies with the RPM model, mice were maintained according to practices prescribed by the University of Utah’s Institutional Animal Care and Use Committee.

### Plasmids and Lentiviral Production

The human NOTCH1 intracellular domain (hN1ICD) doxycycline-inducible expression plasmid (pLIX-hN1ICD) was a gift from Julien Sage (Addgene #91897 [Watertown, MA]).^11^ The pLIX_403 vector (a gift from David Root, Addgene #41395) was used as an empty-vector control. Lentiviral vectors were packaged into lentivirus particles by co-transfecting Lenti-X 293T cells (Clontech, Takara Bio, Kusatsu, Shiga, Japan) with pMDLg/pRRE (a gift from Didier Trono, Addgene #12251), pCMV-VSV-G (a gift from Bob Weinberg, Addgene #8454) and pRSV-Rev (a gift from Didier Trono, Addgene #12253). Lentiviral particles were harvested and filtered (0.45 *μ*m) and CDX cells were infected with 1 mL virus containing 12 *μ*g/mL polybrene (Sigma), followed by selection with 1 *μ*g/mL Puromycin (Merck, P8833 [Rahway, NJ]).

### CDX Longitudinal Growth Study

A total of 100,000 viable CDX22P cells in 100 *μ*L 1:1 RPMI: Matrigel were injected subcutaneously into the right flank of twelve 8 to 12 week-old female NSG mice (Charles River Laboratories). Mice were randomized deterministically into four groups when tumors reached 150 to 200 mm^3^, to be removed at 250 mm^3^, 500 mm^3^, 750 mm^3^, or 1000 mm^3^. This avoided bias of the fastest growing tumors into one group and meant that different tumor growth rates were represented in each group. Three tumors were allocated to each of the four size groups. The study was designed to provide 12 tumors at varying sizes to calculate correlations between tumor size and the vasculature. No animals, experimental groups, or data points were excluded from the study. Blinding was not performed during this experiment as it was an exploratory study and not hypothesis testing. Tumors were harvested in ice-cold formalin for FFPE tissue, and each tumor was analyzed as an individual biological replicate. FFPE tissue was analyzed by immunohistochemistry (IHC) for VM vessels and endothelial vessels (see below). Linear regression analysis (n = 12) of VM vessel score or endothelial vessel score versus tumor volume was performed.

### CDX Tumor Perfusion Study

A total of 100,000 viable CDX cells in 100 *μ*L 1:1 RPMI: Matrigel were injected subcutaneously into the right flank of 8 to 12-week-old female NSG mice (Charles River Laboratories). CDX tumors were grown to approximately 750 mm^3^ and mice received an intravenous injection of biotinylated tomato lectin (4 mg/kg, Vector Laboratories, B-1175-1 [Newark, CA]) 1 hour before they were killed by the schedule 1 method. Tumors were excised and processed to FFPE tissue or snap-frozen in liquid nitrogen, followed by immunofluorescence (IF) analysis for intravenous tomato lectin and endothelial vessels (see below).

### IHC and In Situ Hybridization

FFPE CDX tumors, GEMM tumors, and patient biopsy tumors were cut as 4 *μ*m sections and stained by IHC for markers detailed in Supplementary Table 1. All IHC was standardized on a Leica Bond Max or Rx Platform (Leica Biosystems, Wetzlar, Germany) using standard protocol F with Bond Polymer Refine Detection kit (DS9800) (Leica Biosystems) or on a Roche Ventana Ultra with UltraMap DAB IHC Detection kit (760-151) (Roche, Basel, Switzerland), unless otherwise stated. For VM vessel staining, CD31 was automated on the Leica Bond Max using standard protocol F minus hematoxylin. Periodic Acid Schiff (PAS) staining was performed manually by incubation in 4 mg/mL periodic acid (Sigma-Aldrich, #375810) for 5 minutes followed by incubation in Schiff’s fuchsin-sulfite reagent (Sigma-Aldrich, S5133) for 30 minutes in the dark, before incubation in warm water for 4 minutes and rinsing in water until clear. Chromogenic detection of biotinylated intravenous lectin in FPPE tissues was automated on the Leica Bond Rx using standard protocol F with the post-primary mouse link and secondary detection steps substituted for a streptavidin-biotin-horseradish peroxidase (HRP) step (Vectastain Elite ABC-HRP Peroxidase kit, Vector laboratories, #PK-6100).

Multiplex chromogenic IHC staining was performed on the Leica Bond Max using protocol F minus hematoxylin for CD31, followed by REST or SYP using protocol J with Bond Polymer Refine Red Detection Kit (DS9390) (Leica Biosystems) minus hematoxylin and red parts A, B, C, and D. By manual IHC, slides were then incubated with Vector Blue reagent (Vector laboratories, SK-5300) for 30 minutes. PAS staining was performed thereafter. In situ hybridization for mouse REST (RNAscope LS 2.5 probe – Mm-Rest, ACDBio, #316258 [Advanced Cell Diagnostics, Inc., Newark, CA]) was performed on the Leica Bond RX (Leica Biosystems) and developed with Vector Blue chromagen (Vector Laboratories). VM vessel staining on the Bond Max and PAS staining was performed thereafter.

Whole sections were scanned using a Leica SCN400 (Leica Microsystems, Wetzlar, Germany) or Olympus VS120 (Olympus Life Science, Tokyo, Japan), and single-plex chromogenic staining was quantified using HALO (Indica labs, Albuquerque, NM). For VM vessel scoring, VM vessels and endothelial vessels were counted manually in which endothelial vessels are PAS^+^/CD31^+^ and VM vessels are PAS^+^/CD31^−^ structures with a defined lumen, sometimes containing red blood cells. PAS^+^ mouse stromal cells (defined morphologically by distinct PAS staining patterns through the tumor within regions that do not contain SCLC tumor cells) are excluded from scoring and PAS^+^ VM vessels are scored only within regions containing tumor cells. A VM vessel score was determined as the ratio of VM vessels to total vessels and expressed as a percentage. Patient biopsy tumors were analyzed by two independent scorers without knowledge of demographic or outcome data. For quantification of REST-positive VM vessels in multiplex chromogenic IHC, VM vessels were identified and scored as positive if the vessel lumen was surrounded by two or more REST-positive cells.

### Immunoblotting

SCLC cells were lysed on ice with lysis buffer (Cell Signaling Technology, #9803S [Danvers, MA]) containing a cocktail of protease inhibitors (Sigma, #P8340) and phosphatase inhibitors (Sigma, #P0044 and #P5726). Protein concentrations were measured using the bicinchoninic acid protein assay reagent kit (ThermoFisher Scientific, #23225). 20 *μ*g of each protein lysates were separated by sodium dodecyl-sulfate polyacrylamide gel electrophoresis on 4% to 12% gradient gels (NuPAGE, ThermoFisher Scientific, #NP0322) and transferred onto polyvinylidene difluoride membrane (ThermoFisher Scientific, #10617354). Membranes were blocked with 5% milk diluted in tris-buffered saline 0.1% Tween (Sigma-Aldrich) for 1 hour at room temperature and incubated with primary antibodies (Supplementary Table 2) overnight at 4°C, followed by incubation with goat anti-rabbit IgG HRP (P0440801-2 [Agilent Technologies, Santa Clara, CA]), rabbit anti-mouse IgG HRP (P044701-2 [Agilent technologies]), goat anti-rat IgG HRP (ab57057 [Abcam, Cambridge, United Kingdom]) or rabbit anti-goat IgG HRP (Agilent technologies/P044901-2) secondary antibodies (1:5000). Western blots were developed with Western Lightning chemiluminescence reagent plus (#NEL104001EA [Perkin Elmer, Waltham, MA]) and imaged on a ChemiDoc (Bio-Rad, Hercules, CA). All blots were subsequently reprobed for a sample loading control (tubulin or GAPDH) on the same blot. Two to three lysates from independent tumor replicates were run independently on different blots (one representative blot illustrated per experiment).

### In Vitro Tubule Formation Assay

Culture dishes were coated with Growth Factor Reduced Matrigel (#354230 [Corning, Inc., Corning, New York]) and incubated at 37°C for 60 minutes to set. SCLC cells were seeded onto six-well plates at a density of 1.5 x 10^6^ cells and imaged by phase contrast microscopy after 24 hours. For integrin *β*1–blocking antibody experiments, cells were incubated in media containing 10 *μ*g/mL blocking antibody (purified rat anti-human CD29 clone Mab 13, BD Biosciences, #552828 [BD Biosciences, Franklin Lakes, NJ]) or equivalent concentration non-targeting isotype control (purified rat IgG2a *κ* isotype control clone R35-95, BD Biosciences, #553927). For integrin *α*2, *α*10 and *α*11 combination blocking antibody experiments, cells were incubated in media containing 30 *μ*g/mL total blocking antibody (mouse anti-human integrin *α*2 (CD49b, Biolegend, 359302 [Biolegend, San Diego, California]), rabbit anti-human integrin *α*10 (Millipore, AB6030 [MilliporeSigma, Burlington, MA]), mouse anti-human integrin *α*11 (Nanotools, 0518-100/ITGA11-203E1 [Nanotools GmbH, Munchen, Germany]) or matched isotype controls. For Focal adhesion kinase (FAK) inhibitor experiments, cells were incubated in 1 *μ*g/mL PF-271 (Sigma-Aldrich, #PZ0287) or DMSO control for 1 hour, before seeding onto Matrigel-coated dishes. To quantify tubule branching length, 4 to 5 random field of view brightfield images were taken per well and images were analyzed in ImageJ (National Institutes of Health, Bethesda, MD) using the angiogenesis analyzer algorithm. For confocal imaging, cells were labeled with Molecular Probes CellTracker Green CMFSA Dye (C2925, 1 *μ*g/mL) (ThermoFisher Scientific) or CellTracker Deep Red Dye (C34565, 1 *μ*g/mL) (ThermoFisher Scientific) for 30 minutes according to the manufacturer’s instructions, and 1.5 × 10^6^ labeled cells were seeded out onto a 35mm dish coated with Growth Factor Reduced Matrigel (#354230) (Corning) and allowed to form tubules. Tubules were imaged on a Leica TCS SP8 confocal microscope (Leica Microsystems) using a 25× water lens and z-stacks (40–150 *μ*m) were reconstructed using Imaris Imaging software (Oxford Instruments, Abingdon, United Kingdom).

### Cell Viability Assays

Adherent cells in 100 *μ*L of media were seeded in triplicate in a 96-well plate (1 × 10^3^ cells per well) and cells were allowed to recover for 24 hours. A total of 100 *μ*L of PF-271 (final concentrations between 0 *μ*M and 100 *μ*M) was added to each well, tested in triplicate, and the plate incubated for 24 hours. The number of viable cells was determined using the CellTiter-Glo luminescent assay (#G7570 [Promega, Madison, WI]) in which 20 *μ*L of CellTiter-Glo Reagent (Promega) was added to each well, incubated while shaking for 5 minutes to lyse cells and incubated for a further 30 minutes to stabilize the signal before reading luminescence on a FLUOStar Omega plate reader (BMG Labtech, Ortenberg, Germany). Half maximal inhibitory concentration values were calculated using GraphPad Prism Software version 7 (Dotmatics, Boston, MA), with luminescence normalized to the control wells (0 *μ*M drug) and plotted against drug concentration in *μ*M.

### Immunofluorescence

Cryosectioned CDX tumors with intravenous tomato lectin were fixed with 4% paraformaldehyde (PFA) for 10 minutes, blocked with 1% bovine serum albumin (BSA), 0.3 M glycine, 0.1% Triton X-100 in phosphate-buffered saline (PBS) for 30 minutes and incubated with anti-murine CD31 (1:1000, ab124432 [Abcam]) and anti-human mitochondria (1:250, ab92824 [Abcam]) for 1 hour at room temperature, followed by goat anti-rabbit AlexaFluor-488 (1:1000, #A-11034 [Thermo Fisher Scientific]), goat anti-mouse AlexaFluor-647 (1:1000, #A-21235 [ThermoFisher Scientific]) and streptavidin-PE (1:100, Biolegend 405203 [BioLegend]) secondary antibodies overnight at 4°C. Tissues were mounted and whole sections were scanned on an Olympus VS120 at 20×.

For multiplex immunofluorescence (IF) in CDX FFPE tumors, tissues were cut as 4 *μ*m sections and automated IF was performed on a Leica Bond Rx Platform at room temperature using the Opal 4-Color Automation IHC Kit (#NEL800001KT [PerkinElmer]). Tissue sections were blocked with 3% hydrogen peroxide (Sigma-Aldrich, H1009) for 10 minutes to block endogenous peroxidase activity, followed by 10% casein solution (Vector Laboratories, #SP-5020) for 10 minutes to block non-specific antibody binding. Slides were stained with primary antibody (CD31 or VCAM1) followed by Dako envision+system HRP-conjugated secondary antibody (Dako, #K4003 [Agilent]) for 30 minutes, followed by incubation with Opal Tyramide-fluorophore (PerkinElmer, OPAL650, OPAL570 or OPAL520 1:200) for 10 minutes. For detection of more than one epitope, tissues were heat inactivated after the tyramide-fluorophore incubation step, then blocked, and probed with another primary antibody as above and incubated with a different tyramide-fluorophore. Biotinylated intravenous lectin was detected with the Vectastain Elite ABC-HRP peroxidase kit (Vector Laboratories, #PK-6100) followed by an Opal tyramide-fluorophore. Tissues were counterstained with nuclear 4’,6-diamidino-2-phenylindole (DAPI) (0.1 *μ*g/mL, #10184322 [Thermo Fisher Scientific]) for 10 minutes, slides were mounted in Molecular Probes Pro-Long Gold Antifade (Thermo Fisher Scientific) and whole sections were scanned on an Olympus VS120 at 20X.

For human mitochondria IF 8-well Millicell slides (Millipore #PEZGS0816) were coated with Growth Factor Reduced Matrigel (Corning #354230) and seeded with cells at a density of 1.5 × 10^6^ mL^−1^. After 24 hours, cells were fixed with 4% PFA for 30 minutes and permeabilized in 1% BSA, 0.3 M glycine, and 0.1% Triton X-100 in PBS for 1 hour. Fixed cells were incubated with anti-human mitochondria antibody (Abcam, ab92824) at 1:250 dilution overnight at 4°C and anti-mouse AlexaFluor-555 secondary antibody (1:1000, #A-21424 [Thermo Fisher Scientific]) for 1 hour at room temperature, followed by nuclear DAPI (0.1 *μ*g/mL). Cells were mounted and imaged by fluorescence microscopy.

For tomato lectin and integrin *β*1 IF, 35 mm petri dishes were coated with Growth Factor Reduced Matrigel (Corning #354230) and seeded with cells at a density of 1.5 × 10^6^ mL^−1^. After 3 days, cells were fixed with 4% PFA for 30 minutes and permeabilized in 1% BSA, 0.3 M glycine, and 0.1% Triton X-100 in PBS for 1 hour. Fixed cells were incubated with integrin *β*1– fluorescein isothiocyanate (CD29-FITC, Beckman Coulter, IM0791U, 1:25 [Beckman Coulter, Brea, CA]) and tomato lectin biotinylated (1:250, Vector Laboratories, B-1175-1) for 1 hour at room temperature followed by APC-Streptavidin secondary (1:500, Biolegend, 405207) and nuclear DAPI (0.1 *μ*g/mL, Fisher Scientific, #10184322). Cells were imaged on a Leica TCS SP8 confocal microscope (Leica Microsystems) using a 25× water lens and z-stacks (40–150 *μ*m) were reconstructed using Imaris Imaging software (Oxford Instruments).

### Reverse Transcription-qPCR

RNA was isolated from CDX cells using the RNeasy mini kit (Qiagen, #74106 [Qiagen, Hilden, Germany]) according to Qiagen recommendations. Copy DNA synthesis was performed with the high-capacity copy DNA Reverse Transcription Kit (Thermo Fisher Scientific, #4368814). Reverse transcription quantitative polymerase chain reaction was performed using Taqman (Thermo Fisher Scientific) gene expression master mix and gene expression assays for *ASCL1* (Hs00269932_m1), *SYP* (Hs00300531_m1), *NCAM* (Hs00941830_m1), *CHGA* (Hs00900370_m1), *MYCL* (Hs00420495_m1), *HEY1* (Hs05047713_s1), *REST* (Hs05028212_s1), *YAP1* (Hs00902712_g1), *FOXC2* (Hs00270951_s1), *MYC* (Hs00153408_m1), *ATOH1* (Hs00944192_s1), *NEUROD1* (Hs01922995_s1), and *ACTB* (Hs01060665_g1) according to the manufacturer’s recommendations. Data were analyzed with the delta-delta cycle threshold method by normalizing to *ACTB* housekeeping gene.

### RNAseq and Transcriptomic Analysis

RNA was extracted from 3 to 6 independent replicate tumors per CDX and RNAseq was performed as previously described.^8^ Transcriptomic analysis was performed with amendments to the previously described alignment (NF-core RNAseq pipeline with Spliced Transcripts Alignment to a Reference or STAR aligner [Dobin et al.]^24^) and annotation (mapped to GRCh38 assembly [Ensembl version 99]).^8^ CDX NE and non-NE cells were cultured on plastic and Matrigel for 24 hours, followed by RNA extraction (RNeasy mini kit, Qiagen, #74104) and sequencing. Data were aligned using STAR^24^ to GRCh38 Ensembl version 99^25^ as part of the RNAseq pipeline from NF-core.^26^ Aligned reads were filtered to remove mouse contamination reads using the bamcmp algorithm^27^ before being mapped to genomic annotation.^28^ Downstream analysis was performed in R software (R core team, Vienna, Austria).^29^ Differentially expressed genes were called using DESeq2,^30^ log2 fold change were shrunk using the “ashr” transform^31^ and visualized using the EnhancedVolcano package (Bioconductor). Gene set enrichment analysis was performed using Generally Applicable Gene-Set Enrichment.^32^ For visualizations, the raw counts were transformed by means of the variance stabilizing transform in DESeq2.

### LC-MS/MS Protein Preparation

Two independent GEMM RBL2 *Hes1* reporter lines were separated into NE (GFP^−^) and non-NE (GFP^+^) subpopulations and cells were cultured on plastic and Matrigel for 24 hours, followed by protein lysate preparation and protein concentration quantification (bicinchoninic acid assay); performed in technical triplicate. The sample volume was adjusted to 50 *μ*L by adding more buffer or concentrating using a speed vacuum. 50 *μ*L of 8 M urea (Sigma-Aldrich) was added to each sample and protein disulfide bonds were reduced with 5 *μ*L of 200 mM Tris(2carboxyethyl) phosphine (Sigma-Aldrich) solution and incubated at room temperature for 1.5 hours. Reduced disulfide bonds were capped by adding 7.5 *μ*L of 200 mM iodoacetamide (Acros Organics, ThermoFisher Scientific) solution and incubating for 45 minutes at room temperature in the dark. After incubation, samples were de-salted and trypsin digested after a mini S-trap column protocol provided by the manufacturer (ProtiFi, Fairport, New York). Briefly, 100 *μ*L of 10% sodium dodecyl-sulfate solution, 10 *μ*L of 12% aqueous phosphoric acid, and 1.4 mL of binding buffer were added to the samples, in the order described in the protocol, and vortexed. Acidified lysates were loaded onto S-trap mini spin columns in three aliquots of 500 *μ*L and centrifuged at 4,000 *g* for 60 seconds, collecting the flow-through, until all the lysate had passed through. The flow-through was reloaded again as described above. S-Trap columns were washed with 400 *μ*L binding buffer three times, transferred to a new 2.0 mL Eppendorf tube, and S-trap columns incubated with 150 *μ*L trypsin enzyme digestion buffer (1:30 trypsin enzyme (Thermo Fisher Scientific): protein by weight) overnight at 37°C. Tryptic peptides were eluted from the S-trap column by means of centrifugation at 1000 *g* for 60 seconds and for shotgun proteomics analysis 5 *μ*L of the tryptic digest solution from each sample was dried down using a speed vacuum and reconstituted back into solution by adding 12 *μ*L of 0.1% formic acid in water. The 3 *μ*L injections of each sample were analyzed by liquid chromatography-tandem mass spectrometry (LC-MS/MS) in triplicate.

### LC-MS/MS Analysis

Shotgun proteomics was performed on an LTQ-Orbitrap Elite mass spectrometer (Thermo Fisher Scientific) connected to a Dionex UltiMate RS 3000 nano-LC (Thermo Fisher Scientific). Peptides were loaded onto a C18 trap column (Acclaim PepMap, 100 A 5 *μ*m particle size) (Thermo Fisher Scientific) at a flow rate of 5 *μ*L/min in solvent A (0.1% formic acid in water) and desalted for 10 minutes. Tryptic peptides were then separated by a reversed-phase C18 analytical column (25-cm long, packed with Magic AQ C18 resin) (Michrom Bioresources, Auburn, CA). Peptides were eluted by changing the concentration of solvent B (0.1% formic acid in acetonitrile) from 2% (first 10 min), to 35% (over 100 min), and 85% (next 2 min followed by 5 min hold at 85%). Eluted peptides were subjected to MS1 and MS/MS on the mass spectrometer. The MS1 mass resolution was set to 60,000 with a scan range of 400 to 1800 m/z. The top 10 most abundant ions in each MS1 scan were selected for collision-induced dissociation. Dynamic exclusion was set for 30 seconds.

### Total Protein Raw Data Analysis

MS raw files from the shotgun proteomics analysis were searched against the Swiss-Prot mouse database using Byonic (Protein Metrics, Cupertino, CA) software. Quantitative information was extracted from MS1 spectra of all identified peptides using an in-house R script on the basis of MSnbase package^33^ and integrated from the spectrum to protein level using the WSPP (weighted spectrum, peptide, and protein) model^34^ with the SanXoT software package.^35^ In summary, every scan *x*_*qps*_ = log_2_*A/B* was calculated using the area under the curve of the extracted ion current coming from group 1 and group 2. The statistical weight *w*_*qps*_ of each scan was calculated as the maximum area under the curve of the pair of samples to compare. The log2-ratio of every peptide (*x*_*qp*_) was calculated as the weighted average of its scans, whereas the quantification of each protein (*x*_*p*_) was the weighted average of its peptides, and the grand mean ($\bar{x}$) as the weighted average of all the protein measures. The variances at the scan, peptide, and protein levels, and protein-abundant changes were determined only with non-modified peptides.

### Proteomics Downstream Data Analysis

For analysis of shotgun proteomics, Matrigel contaminants were excluded, and *t* tests were performed between the non-NE Matrigel and non-NE plastic samples. Significant differentially expression proteins were then compared with the NE Matrigel samples to generate non-NE Matrigel specific (VM, network-forming) up-regulated and down-regulated proteins. Fold change was plotted against −log(q-values) in GraphPad prism to generate volcano plots. Gene ontology (GO) analysis was performed using the Database for Annotation, Visualization, and Integrated Discovery functional annotation tool online (https://david.ncifcrf.gov/)^36^ (Laboratory of Human Retrovirology and Immunoinformatics).

### ECM Adherence Assays

ECM adherence assays were performed as previously described.^37^ Dishes were incubated with 1 mg/mL collagen 1, 1 mg/mL laminin, or PBS overnight at 4°C, followed by incubation with heat denatured BSA solution (10 mg/mL, Sigma-Aldrich, A3608) for 30 minutes, before rinsing in PBS and plating cells. Plates were fixed with 5% glutaraldehyde for 30 minutes and cells were counterstained with 1% crystal violet solution in distilled water and imaged. The surface area of cells was determined in ImageJ by automatic cell detection after thresholding. For integrin *β*1–blocking antibody experiments, cells were incubated in media containing 10 *μ*g/mL blocking antibody or equivalent concentration non-targeting isotype control, as above.

### ECM Remodeling Assays and In Vitro Staining

Cells on Matrigel were fixed with 4% PFA for 10 minutes and washed once in PBS. For collagen assessment by picrosirius red (PSR) staining, cells were incubated for 1 hour at room temperature with PSR staining solution before rinsing twice in acetic acid solution (picrosirius red stain kit, Abcam, ab150681) and imaging. For glycoprotein assessment, cells were incubated for 5 minutes in 0.5% periodic acid (Sigma-Aldrich, 375810), rinsing wells twice in PBS, staining with Schiff’s fushin-sulfite reagent (Sigma-Aldrich, S5133) for 15 minutes, rinsing extensively in PBS, and imaging by light microscopy.

### Flow Cytometry

Flow cytometry for integrin expression within CDX cells *ex vivo* was performed on a BD LSRFortessa (BD Biosciences). Cells were accutased, passed through a 70 *μ*m strainer to generate a single cell population, and washed in PBS 0.1% BSA. Single cells were incubated with rat anti-human integrin *β*1 (CD29, BD biosciences, 552828), mouse anti-human integrin *α*2 (CD49b, Biolegend, 359302), rabbit anti-human integrin *α*10 (Millipore, AB6030), mouse anti-human integrin *α*11 (Nanotools, 0518-100/ITGA11-203E1) or isotype controls for 30 minutes at room temperature. Cells were washed three times in PBS 0.1% BSA and incubated with appropriate secondary antibody (1:1000) for 30 minutes at room tempera-ure. Cells were washed three times in PBS 0.1% BSA before flow cytometry analysis. Spectral compensation was performed and cytometry gating strategies were set according to fluorescence minus one-stained CDX cells.

### Dye-Quenched Collagen Assays

To assess collagen cleavage in vitro, cells were incubated for 72 hours in media or media containing 20 *μ*g/mL dye-quenched (DQ) collagen (DQ Collagen, type I from Bovine Skin, Fluorescein Conjugate, D12060) (Invitrogen, Waltham, MA) and fluorescence was measured on a plate reader and normalized to media-only containing wells with and without DQ collagen. To assess DQ collagen remodeling during VM formation in vitro, DQ collagen was added to Matrigel at a concentration of 100 *μ*g/mL, and a VM assay was performed as described above. Brightfield and fluorescence images were acquired, processed (background removal standardized to all images within the same experiment and green false color on fluorescence images), and overlayed in ImageJ.

### Quantification and Statistical Analysis

Statistical tests were performed using GraphPad Prism or Excel. Error bars illustrate the mean (± standard error of the mean [SEM]) unless otherwise specified. Significance was determined by the Student’s two-tailed unpaired *t* tests with 95% confidence intervals and *p* values, 0.05 considered statistically significant unless otherwise indicated. All statistical details are further described in respective figure legends.

## Results

### CDX and GEMMs Are Tractable Models to Study VM

**Figure 1.**
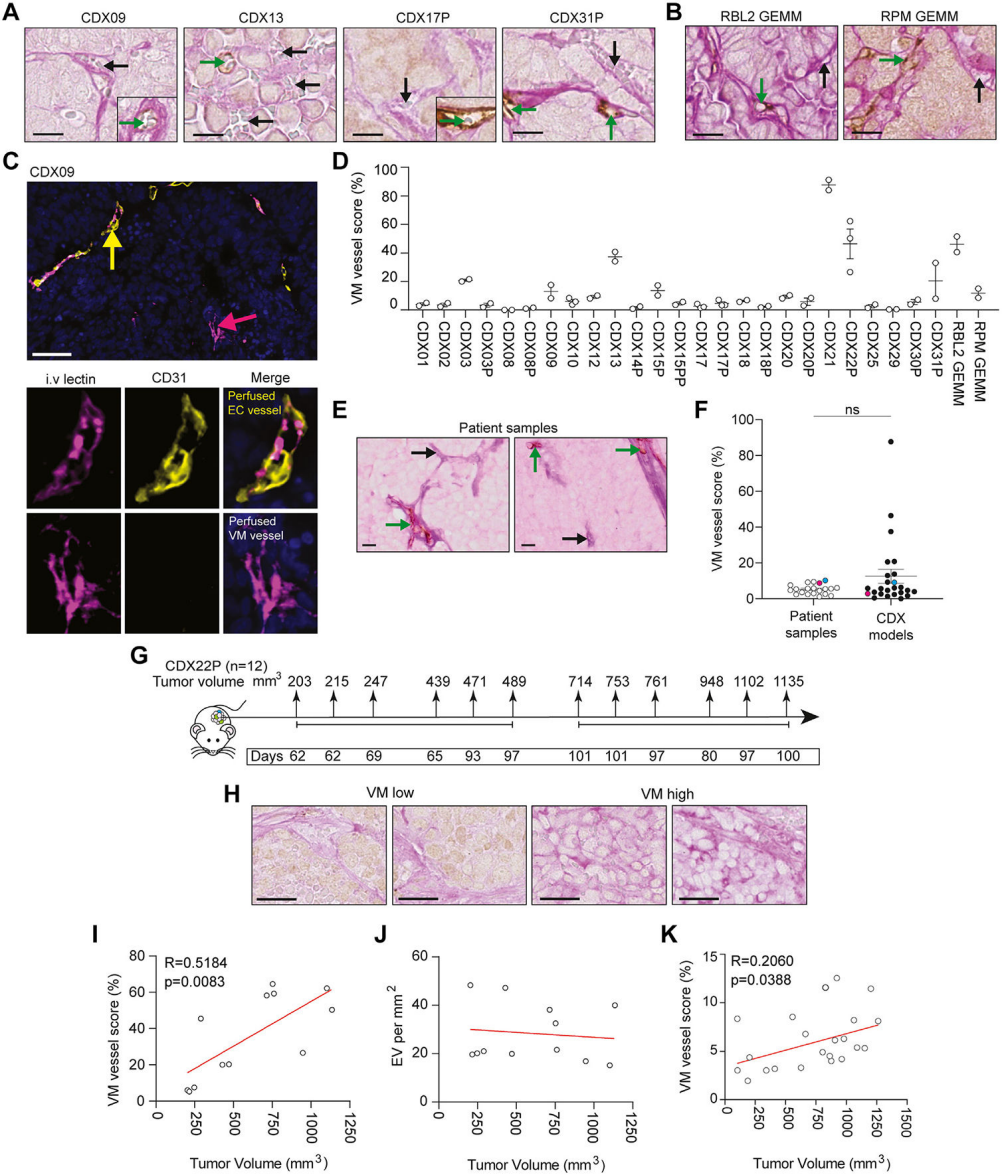
VM in SCLC CDX and GEMM. (*A*) IHC of VM vessels (PAS^+^/CD31^−^, pink, black arrows) and endothelial vessels (PAS^+^/CD31^+^, brown, green arrows) in CDX. Scale bars, 200 *μ*m. CDX models were generated from patient CTCs at pre-chemotherapy baseline or at post-treatment disease progression time points (designated P). (*B*) IHC of VM vessels (PAS^+^/CD31^−^, pink, black arrows) and endothelial vessels (PAS^+^/CD31^+^, brown, green arrows) in RBL2 (*Trp53^fl/fl^*/*Rb1^fl/fl^*/*Rbl2^fl/fl^*)^22^ and RPM (*Trp53^fl/fl^*/*Rb1^fl/fl^*/*Myc^LSL/LSL^*)^23^ GEMMs. Scale bars 200 *μ*m. (*C*) Representative IF image of a perfused endothelial (EC) vessel (CD31^+^/intravenous tomato lectin^+^, yellow arrow) and a perfused VM vessel (CD31^−^/intravenous tomato lectin^+^, pink arrow) within a CDX09 tumor that was harvested after intravenous tomato lectin injection. Single-channel IF for CD31 (yellow) and intravenous tomato lectin (pink) illustrated with merged multiplex on the right in which nuclei are blue (DAPI stain). Scale bars 50 *μ*m (left panel). (*D*) VM vessel score (% VM vessels relative to total vessels [VM + endothelial]) in CDX and GEMM (n = 2-3 independent tumor replicates). (*E*) IHC of VM vessels (PAS+/CD31−, pink, black arrows) and endothelial vessels (PAS+/CD31+, brown, green arrows) in SCLC patient biopsy samples. Scale bars 10 *μ*m. (*F*) VM vessel score (% VM vessels relative to total vessels [VM + endothelial]) in SCLC patient biopsy samples (white circles) versus CDX models (black circles). CDX12 (blue) and CDX25 (pink) have a matched patient tumor biopsy generated from CTCs from those patients. (*G*) Experimental design to assess VM at increasing tumor volumes. A total of 12 mice were killed by the schedule 1 method between 8 and 15 weeks with tumor sizes ranging from 203 mm^3^ to 1135 mm^3^. (*H*) IHC of VM vessels (PAS^+^/CD31^−^, pink, black arrows) in independent CDX22P tumors ranging from 203 mm^3^ to 1135 mm^3^. Scale bar 25 *μ*m. (*I*) Pearson correlation of VM vessel score versus tumor volume in CDX22P. Each circle represents an independent tumor replicate. (R = 0.5184, *p* = 0083). (*J*) Pearson correlation of EV per mm^2^ versus tumor volume. Each circle represents an independent tumor replicate. (*K*) Pearson correlation of VM vessel score versus tumor volume. Each circle represents an independent tumor replicate. (R = 0.2060, *p* = 0.0388). CDX, circulating tumor cell-derived explant; CTC, circulating tumor cell; DAPI, 4’,6-diamidino-2-phenylindole; EC, endothelial cell; EV, endothelial vessels; GEMM, genetically engineered mouse model; IF, immunofluorescence; IHC, immunohistochemistry; PAS, Periodic Acid Schiff stain; VM, vasculogenic mimicry.

VM vessels were scored using PAS+/CD31− IHC in 25 CDX models^8^ (Fig. 1A-D and Supplementary Table 3), including the previously unpublished CDX21 (Supplementary Fig. 1) and the RBL2 (*Trp53^fl/fl^*/*Rb1^fl/fl^*/*Rbl2^fl/fl^*) and RPM (*Trp53^fl/fl^*/*Rb1^fl/fl^*/*Myc^LSL/LSL^*) GEMMs^22,23^ (Fig. 1B). VM vessels had lumens that frequently contained red blood cells (e.g., Fig 1A and B black arrows), suggesting perfusion and endothelial vessel connectivity (CD31+ brown stain) (e.g., Fig. 1A and B, green arrows). Perfusion through VM vessels was also inferred by intravenous injection of tomato lectin into mice harboring CDX09 tumors, which labels glycoproteins lining the inside of functional vessels carrying blood.^38,39^ IF for intravenous tomato lectin (pink) and CD31 (yellow) within CDX09 tumors (Fig. 1C and Supplementary Fig. 2) revealed perfused, hollow VM vessels (tomato lectin+/CD31−) (pink arrow) lined by human tumor cells and perfused, hollow endothelial vessels (tomato lectin+/CD31+) (yellow arrow), providing evidence that VM vessels that support blood flow are present in CDX tumors. CDX VM vessel score (% VM vessels of total VM plus endothelial vessels) ranged from 0% to 87% (median 5%) (Fig. 1D). Only 2 of 25 models (CDX08, CDX29) contained no detectable VM vessels (Fig. 1D). A cohort of limited and extensive-stage SCLC patient biopsy samples (n = 20) (clinical characteristics summarized in Supplementary Table 4) were stained with human anti-CD31 and PAS to investigate the prevalence of VM in clinical specimens. PAS+/CD31− VM vessels (Fig. 1E, black arrows) and PAS+/CD31+ endothelial vessels (Fig. 1E, green arrows) were present in all patient specimens, in which VM vessel score ranged from 1% to 10% (median 5%) and was comparable to CDX models (Fig. 1F). Two CDX models (CDX12, blue; CDX25, pink) had a matched patient tumor biopsy in which analysis of VM and endothelial vessels was possible in the CDX tissue and their respective donor patient tumors and demonstrated that VM is present, and comparable between the patient sample and the matching CDX model (Fig. 1F). Because VM correlated with accelerated tumor growth in an SCLC xenograft^21^ we assessed whether VM vessel prevalence increased with tumor size. Twelve CDX22P tumors (with robustly quantifiable and reproducible VM) were harvested over 8 to 15 weeks and VM vessels were evaluated in tumors ranging from 203 to 1135 mm^3^ (Fig. 1G). PAS+/CD31− VM vessels (pink) were present in all tumors (Fig. 1H) and VM vessel score positively correlated with tumor volume *in vivo* (*p* = 0.0083) (Fig. 1I) whereas host endothelial vessel density remained stable (Fig. 1J). The positive correlation between tumor volume and VM vessel score was also observed in 21 CDX31P tumors ranging from 200 to 1262 mm^3^ (*p* = 0.0388) (Fig. 1K), confirming our findings in an additional CDX model. Overall, these data indicate that CDX and GEMMs are tractable models for VM studies and that VM vessel networks are composed of perfusable, tubular structures that support tumor growth.

### VM Vessels Lack NE Differentiation Markers and Co-localize With REST^pos^ Non-NE Cells

**Figure 2.**
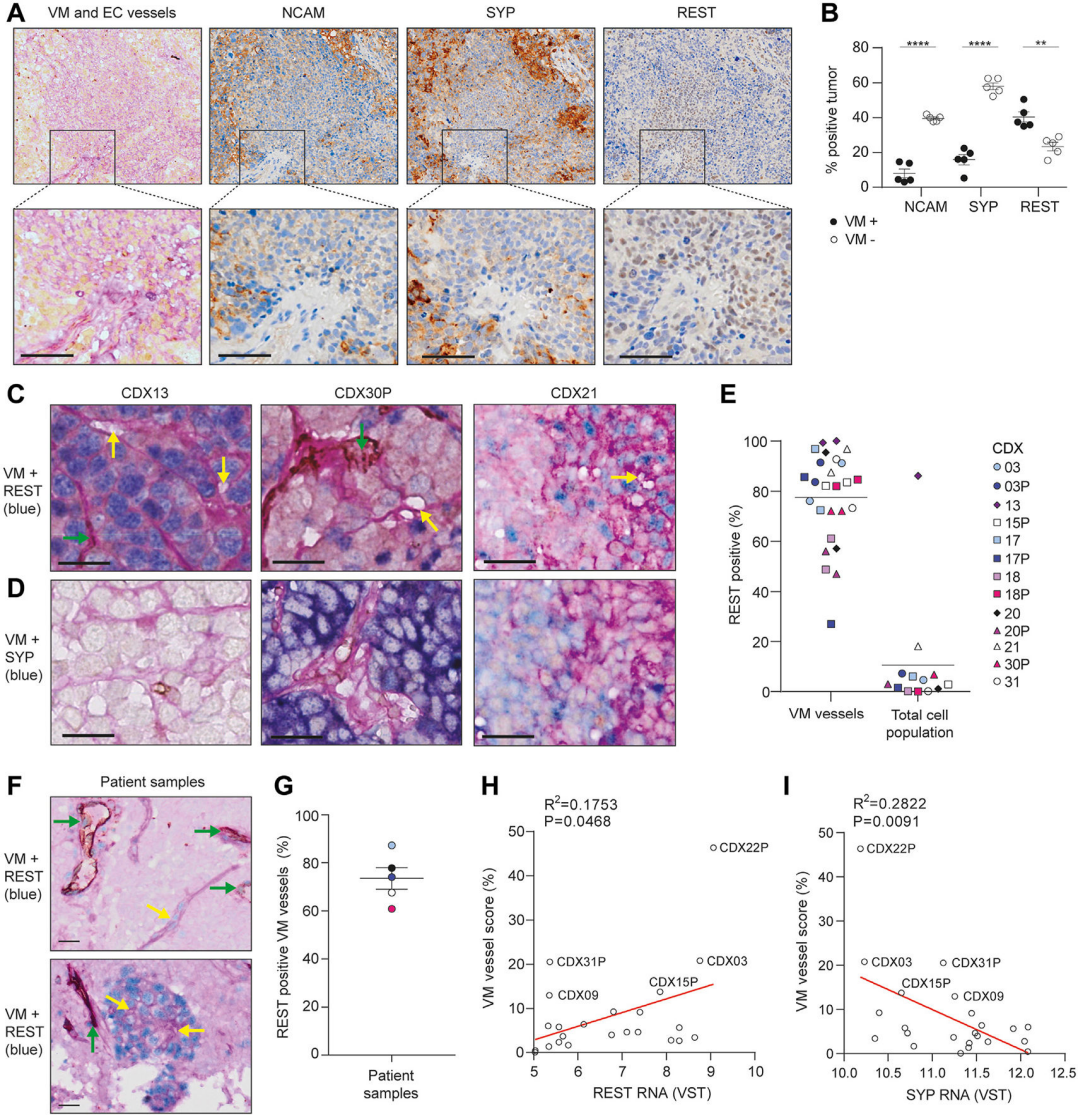
VM vessels co-localize with REST^+^ non-NE cells. (*A*) IHC in serial CDX21 tissue sections for VM vessels (PAS^+^/CD31^−^, pink), endothelial vessels (PAS^+^/CD31^+^, pink+brown), REST, SYP and NCAM (brown). Scale bars, 50 *μ*m. (*B*) Quantification of SYP, NCAM, and REST expression in VM-positive (black circles) and VM-negative (open circles) regions of CDX identified in (A). Each circle represents a region that was defined and quantified, and two independent mice were analyzed (*n* = 2 mice, *n* = 5 regions). Data are mean (± SEM). (*C, D*) Multiplex IHC showing VM vessels (PAS^+^/CD31^−^, yellow arrows), endothelial vessels (PAS^+^/CD31^+^, brown, green arrows), and REST (blue, in C) or SYP (blue, in D). Scale bars, 100 *μ*m. (*E*) Percentage of VM vessels co-localized with REST (REST^+^ VM vessels) (n = 2 mice per CDX). Where the total REST expression for each CDX was derived from.^8^ Tumor sections from three independent mice per CDX were analyzed and the mean REST expression plotted. (*F*) Multiplex IHC illustrating VM vessels (PAS^+^/CD31^−^, yellow arrows), endothelial vessels (PAS^+^/CD31^+^, brown, green arrows), and REST (blue) in SCLC patient biopsy samples. Scale bars, 20 *μ*m. (*G*) Percentage of VM vessels co-localized with REST in SCLC patient biopsy samples. n = 5 (<4% VM vessel score and <20 VM vessels per section; VM vessel scores are 9% (light blue), 5% (black), 4% (dark blue), 6% (white), and 9% (pink). (*H*) Pearson correlation of VM score versus *REST* transcript expression in CDX (R = 0.1753, *p* = 0.0468). The average *REST* expression is illustrated for three independent tumors per CDX.^8^ (*I*) Pearson correlation of VM score versus *SYP* transcript expression in CDX (R = 0.2822, *p* = 0.0091). The average *SYP* expression is illustrated for three independent tumors per CDX.^8^ CDX13 was removed from the analyses in *H* and *I* as there was a high level of VM vessels and REST expression in the tumor, which may bias the analysis. **p < 0.01, ****p < 0.0001 two-tailed unpaired Student’s *t* test. CDX, circulating tumor cell-derived explant; IHC, immunohistochemistry; NE, neuroendocrine; PAS, Periodic Acid Schiff stain; SEM, standard error of the mean; VM, vasculogenic mimicry.

The majority of CDX contain mostly NE cells with a minority of non-NE cells. We sought to determine whether one or both phenotypes were VM-competent. CDX21 contains discrete VM vessel-positive and VM vessel-negative regions (Fig. 2A) for marker co-localization analysis. In serial tissue sections of CDX21 stained with NE markers SYP or NCAM and the non-NE marker REST, cells within VM-positive regions versus VM-negative regions had significantly reduced expression of SYP (15.9% versus 5hN1ICD8.0%, *p* < 0.0001) and NCAM (8.1% versus 39.6%, *p* < 0.0001), and significantly higher expression of REST (40.4% versus 23.4%, *p* = 0.002) (Fig. 2B). Multiplex chromogenic IHC for VM vessels and REST (Fig. 2C) or SYP (Fig. 2D) in 13 CDX models, representing a range of VM vessel scores (Fig. 1C) and NE to non-NE cell ratios^8^ revealed that VM vessel lumens were surrounded by cells with REST-positive nuclei (Fig. 2C yellow arrows) lacking SYP expression (Fig. 2D). Despite the low percentage of total REST-expressing cells (<10%), the majority of VM vessels were lined with REST-positive cells (mean 74%, range: 27%–99%) (Fig. 1E). CDX13, the only non-NE POU2F3 subtype CDX^8^ was the only model with REST expressed throughout the tumor, in which VM vessels were abundant (VM vessel score 38%) (Fig. 1C) and co-localized with REST (Fig. 2C and E). The association between VM vessels and the non-NE marker REST was corroborated in 5 SCLC patient biopsy samples (clinical characteristics summarized in Supplementary Table 4) stained with human anti-CD31, human anti-REST and PAS (Fig. 2F), in which 73.5% of VM vessels co-localized with REST-positive nuclei (Fig. 2G and F yellow arrows). REST-positive nuclei also co-localized with CD31^+^ human endothelial vessels (Fig. 2F green arrows) and exhibited the same pattern of staining as VM vessels. In CDX bulk RNAseq data,^8^ VM vessel score correlated positively with *REST* (Fig. 2H) (R = 0.1753, *p* = 0.0468) and negatively with SYP (Fig. 2I) (R = 0.2822, *p* = 0.0091). In GEMM tumors, cells lining VM vessels co-localized with *REST* transcript detected by RNA in situ hybridization (absolute quantification of co-localization was not feasible by in situ hybridization) (Supplementary Fig. 3). Together, these co-localization data reveal mutual exclusivity between VM vessels and NE cells and identify the minority non-NE cell subpopulation as VM-competent in human and mouse SCLC tumors.

### NOTCH Signaling Drives a NE to Non-NE Transition That Enables Network Formation Consisting of Hollow Tubules in CDX Ex Vivo Cultures

**Figure 3.**
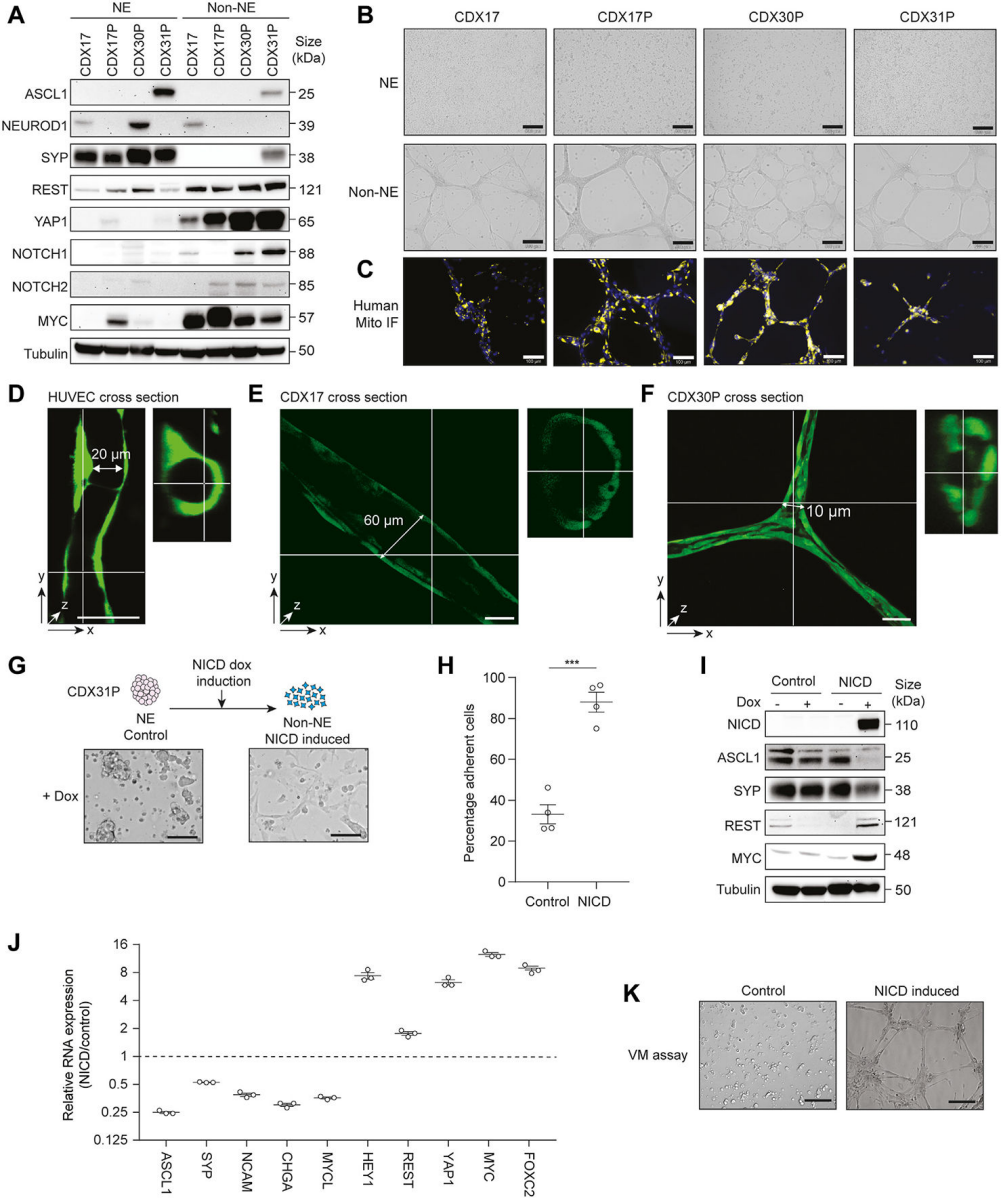
Non-NE CDX cells *ex vivo* are VM-competent and require NOTCH signaling. (*A*) Representative immunoblots of CDX NE and non-NE cell lysates. There are two to three independent replicate tumors per CDX. Tubulin loading control was run subsequently for each marker illustrated on the same blot. (*B*) Representative brightfield images of tubule-forming assay with CDX NE and non-NE cells. There were two to three independent replicate tumors per CDX. Scale bars, 500 *μ*m. (*C*) Representative immunofluorescence of CDX non-NE cells in tubule-forming assay stained for human mitochondria (yellow) and nuclear DAPI (blue) in (*B*). There were two to three independent replicate tumors per CDX. Scale bars, 100 *μ*m. (*D-F*) Representative images of HUVECs (*D*), CDX17 (*E*), and CDX30P (*F*) non-NE cells labeled with Cell Tracker Green forming hollow tubules when grown on Matrigel for 72 hours. Confocal microscopy images are illustrated after Z-stack software reconstruction (Imaris). Tubule length and diameter dimensions are illustrated, scale bars 50 *μ*m. (*G*) Representative images of empty-vector control and NICD expressing CDX31P cells three weeks after dox induction. Scale bars, 250 *μ*m. (*H*) Percentage of adherent cells in control versus NICD-expressing CDX31P cells three weeks after dox induction in the suspension cells (four replicates from one tumor sample). Data are mean (± SEM) (***p < 0.001 two-tailed unpaired Student’s *t* test. (*I*) Representative immunoblots in control and NICD CDX31P cells with or without dox. Tubulin loading control was run subsequently for each marker illustrated on the same blot. (*J*) Reverse transcription quantitative PCR analysis of NE (*ASCL1, SYP, NCAm, cHgA, MYCL*) and non-NE (*HEY1, REST, YAP1, FOXC2, MYC*) markers in control versus NICD-expressing CDX31P cells. Mean values are illustrated (black lines) in which each circle represents one independent analysis, error bars are (± SEM). (*K*) Representative images of tubule-forming assay with control and NICD CDX31P cells 3 weeks after dox induction. Scale bars, 200 *μ*m. There are three independent replicate tumors. CDX, circulating tumor cell-derived explant; DAPI, 4’,6-diamidino-2-phenylindole; dox, doxycycline; HUVECs, human umbilical vein endothelial cells; NE, neuroendocrine; NICD, NOTCH 1 intracellular domain; PCR, polymerase chain reaction; SEM, standard error of the mean; VM, vasculogenic mimicry.

*Ex vivo* cultures of SCLC CDX recapitulate the phenotypic and molecular heterogeneity of CDX *in vivo*,^40^ containing mixtures of suspension (NE) and adherent (non-NE) cells.^41^ Separated adherent and suspension cultures were established from four CDX and their respective NE and non-NE phenotypes were confirmed by marker expression (Fig. 3A). CDX31P is an ASCL1 subtype whereas CDX17/17P and CDX30P are ATOH1 subtypes (no ATOH1 antibody is available) and CDX17 and CDX30P NE cells co-express NEUROD1.^8^ In all models, suspension cells express SYP and adherent cells express the non-NE markers REST and YAP1^7,41^ (Fig. 3A). Active NOTCH promotes NE to non-NE phenotype transition in RBL2 and RPM SCLC GEMMs^9,11^ and adherent CDX cells expressed cleaved (active) NOTCH (NOTCH1 or NOTCH2, or both) (Fig. 3A). Adherent cells from all four CDX also expressed MYC, which is associated with NE-low and non-NE SCLC.^10,23^ Collectively, these data validate physical separation to interrogate VM in NE and non-NE subpopulations.

Formation of branching networks by cells on Matrigel is an established in vitro surrogate assay for VM competence.^17,18,21,42^ When NE and non-NE cell subpopulations from these four CDX models were cultured on Matrigel and stained for human mitochondria, only the non-NE adherent human cells formed branching networks (Fig. 3B and C). Similarly, only non-NE cells from both RPM and RBL2 GEMMs formed networks on Matrigel (Supplementary Fig. 4A and B). Human umbilical vein endothelial cells (HUVEC) cells are the archetypal endothelial cell line used to study vasculogenesis and form hollow networks in vitro on Matrigel^43,44^ (e.g., Fig. 3D). When cultured under identical conditions on Matrigel, we asked whether branching networks formed by SCLC models were comparable to the hollow tubules formed by HUVECs, thus inferring functional similarities *in vivo*. To interrogate this, fluorescently labeled CDX non-NE cells were cultured on Matrigel for three days and analyzed by fluorescence confocal microscopy. Three-dimensional reconstruction of confocal microscopy z-stacks exhibited CDX cells form 3D tubules containing a hollow lumen (Fig. 3E and F and Supplementary Fig. 4C and D, in which representative images are illustrated). Tubule length and lumen diameter varied between the three CDX models tested (CDX17, CDX17P, CDX30P) and the average tubule length was 370 *μ*m (range: 200–570 *μ*m) with an average lumen diameter of 28 *μ*m (range: 10–60 *μ*m) compared with 17 *μ*m (range: 14–20 *μ*m) for HUVEC tubules. The CDX tubule diameters were greater than that of a small capillary (3–8 *μ*m in diameter) and would be sufficient to enable the flow of erythrocytes *in vivo* (~6–8 *μ*m in diameter).^45^

NE to non-NE phenotype switching in SCLC GEMMs is driven by NOTCH signaling^9-11^ so we next asked whether NOTCH activation promotes NE to non-NE switching in CDX to enable VM network formation *ex vivo*. We generated CDX31P suspension NE cells with a doxycycline-inducible NICD to drive NOTCH signaling and assessed NE to non-NE transition 3 weeks after NICD induction by quantification of adherent cells. After inducible expression of NICD, 88% of NOTCH-active CDX31P cells became adherent compared with 33% of empty-vector control cells (Fig. 3G and H) (*p* = 0.0002) supporting tumor plasticity rather than solely the pre-existence of non-NE cells in CDX tumors giving rise to non-NE progeny cells. On NOTCH activation, NE marker expression (ASCL1, SYP) was reduced with concomitantly increased non-NE marker expression (REST, YAP1, MYC, CD44) (Fig. 3I and Supplementary Fig. 4E). Expression (by reverse transcription–quantitative polymerase chain reaction) of a larger panel of NE (*ASCL1, SYP, NCAM, CHGA, MYCL*) and non-NE (*REST, MYC, HEY1, YAP1*) markers exhibited reciprocal expression in control versus induced NOTCH-active cells (Fig. 3J). The TF *FOXC2*, recently reported as a driver of VM in multiple cancer types^46^ was also up-regulated 9-fold in NOTCH-active cells (Fig. 3J). When cultured on Matrigel, only NICD-expressing CDX31P cells formed networks whereas control cells that remained NE and grew in suspension did not (Fig. 3K). These data confirm that NOTCH signaling can drive NE to non-NE transition in a human ASCL1 subtype CDX model and that only non-NE cells are VM-competent.

### Non-NE Cells Express Hypoxic, Vascular Endothelial, and Cell-ECM Remodeling Gene Signatures and Are Transcriptionally Primed for VM

**Figure 4.**
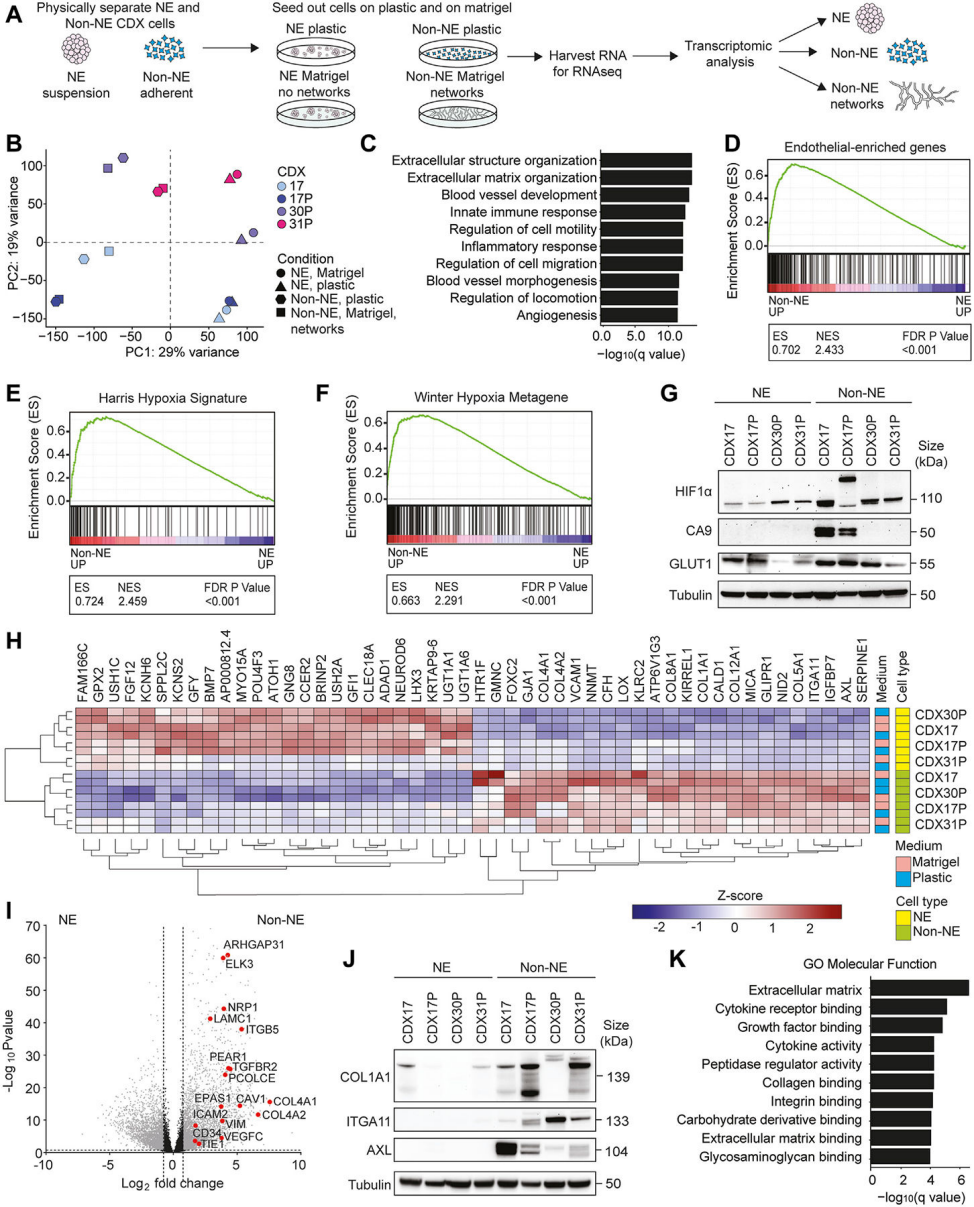
Transcriptomic analysis of network-forming CDX cells. (*A*) Workflow for generation of CDX suspension NE and adherent non-NE cells that were physically separated and cultured on plastic and Matrigel. From these samples, RNA was isolated for RNAseq followed by transcriptomic analysis. (*B*) Principal component analysis of CDX (CDX17, light blue, CDX17P, dark blue, CDX30P, purple, CDX31P, pink) NE and non-NE cell transcriptomes cultured on plastic and Matrigel. Each symbol represents an individual replicate: circles, NE cells on Matrigel, triangles, NE cells on plastic, hexagons, non-NE cells on plastic, squares, non-NE cells on Matrigel (network-forming) per CDX model. (*C*) GSEA of biological processes up-regulated in CDX non-NE cells compared with CDX NE cells. (*D*) GSEA of endothelial enriched genes^47^ in CDX NE and non-NE cell transcriptomes. The endothelial gene set was refined to remove any genes that are expressed in mesenchymal cells.^48^ (*E*) GSEA of Harris hypoxia signature^52^ in CDX NE and non-NE cell transcriptomes. (*F*) GSEA of Winter hypoxia metagene signature^53^ in CDX NE and non-NE cell transcriptomes. (*G*) Representative immunoblots in CDX NE and non-NE cell lysates. There are 2 to 3 independent replicate tumors per CDX. Tubulin loading control was run subsequently for each marker illustrated on the same blot. (*H*) Heatmap of the top 25 up-regulated and down-regulated genes in CDX non-NE cells (green) compared with NE cells (yellow), cultured on either plastic (blue) or Matrigel (pink). (*I*) Volcano plot of differentially expressed genes in CDX non-NE cells compared with NE cells. Significant (fold change >1, −log(qvalue) >1) genes in gray. (*J*) Representative immunoblots of CDX NE and non-NE cell lysates. There are 2 to 3 independent replicate tumors per CDX. Tubulin loading control was run subsequently for each marker illustrated on the same blot (*K*) GO molecular functions up-regulated in CDX non-NE cells compared with CDX NE cells. For GSEA in C, D, E, F, and K, NE and non-NE cells grown on plastic and Matrigel were combined and treated as technical replicates because there were no significant transcriptomic changes identified between these culture conditions. CDX, circulating tumor cell-derived explant; GO, gene ontology; GSEA, gene set enrichment analysis; NE, neuroendocrine; RNAseq, RNA sequencing.

To define molecular processes in SCLC VM, we profiled gene expression by RNAseq in separated NE and non-NE cells from four CDX cultured on plastic or Matrigel (Fig. 4A) and tested the hypothesis that a VM-specific signature would be observed only in non-NE cells forming networks on Matrigel. Principal component analysis revealed the greatest variance (29%) between NE and non-NE cell subpopulations, followed by a 19% variance between CDX models (Fig. 4B). As expected, suspension cell gene expression profiles aligned closely with a published SCLC NE gene signature^10^ (Supplementary Fig. 5), and in non-NE cells there was increased transcription of genes associated with NOTCH pathway activation and down-regulated inhibitory NOTCH pathway ligands (Supplementary Table 5). However, counter to our hypothesis, the clear phenotypic and functional differences between non-NE cells on Matrigel forming networks and those on plastic, incapable of network formation, were not reflected in differential gene expression. This implies that NE to non-NE transition transcriptionally primes cells for VM, but that additional stimuli are required to form vessels. Gene set enrichment analysis (GSEA) of NE and non-NE cells from the four CDX models revealed non-NE cell transcriptomes were enriched for blood vessel development, ECM organization, and cell migration (Fig. 4C) consistent with network formation on Matrigel and endothelial cell behaviors (Figs. 1 and 3). This is substantiated by the relative up-regulation of an endothelial-specific gene set,^47^ that we have refined to remove mesenchymal genes used to implicate epithelial-to-mesenchymal transition,^48^ in non-NE vs NE cells (Fig. 4D and Supplementary Table 6), also in keeping with VM-competent cells in breast and other tumor types,^46^ and the up-regulation of functional vascular protubulogenic genes (e.g. *VEGFC, FLT1, ESM1, TIE1, TEK, CD34*) and blood coagulation cascade genes (e.g. *TFPI, TFPI2, THBD, SERPINE1/2, PLAU*) in the non-NE cells (Supplementary Table 6). Physiological hypoxia, a primary driver of angiogenesis^49^ stimulates VM in several cancer types.^20,50,51^ While non-NE CDX cells harbor hypoxia gene signatures (Fig. 4E and F),^52,53^ network formation occurs in well-oxygenated Matrigel. We reasoned that this paradox might be explained if non-NE cells acquire pseudohypoxic attributes^54^ and confirmed that non-NE cells (in 21% oxygen) exhibit stabilized HIF-1*α* and up-regulation of its downstream effectors GLUT1 or CA9, or both, compared with their NE counterpart cells (Fig. 4G).

The top 25 up-regulated genes (>80 fold) in CDX non-NE cells included cell-cell adhesion receptor *VCAM1*, cell-ECM adhesion receptor *ITGA11*, and multiple fibrillar and basement membrane collagen genes (*COL1A1, COL4A1, COL4A2, COL8A1, COL5A1*, and *COL12A1*) (Fig. 4H). The VM-associated anticoagulant^42^ and SCLC brain metastasis colonization factor^55^ *SERPINE1*, the angiogenesis-associated AXL receptor tyrosine kinase,^56^ master-regulator of VM *FOXC2*^46^ and endothelial-associated genes^57^ were all significantly up-regulated in non-NE compared with NE cells (Fig. 4H and I). Increased expression of COL1A1, ITGA11, and AXL proteins in non-NE CDX cells compared with their NE counterparts was confirmed in *ex vivo* cultures (Fig. 4J). GSEA analysis also implicated processes associated with ECM production, collagen binding, and integrin signaling in the non-NE cells (Fig. 4K). Carbohydrate and glycosaminoglycan binding molecular functions were enriched in non-NE cells (Fig. 4K), supporting the histopathology of VM vessels *in vivo* that are lined by PAS+ glycoprotein basement membrane and marked by lectin when intravenously injected (Fig. 1). Collectively, our RNAseq data revealed that non-NE CDX cells are transcriptionally primed for VM given a conducive microenvironment and implicate cell-cell and cell-ECM interactions as VM-enabling processes.

### Changes in the Cell Adhesion Proteome During Non-NE Cell VM

**Figure 5.**
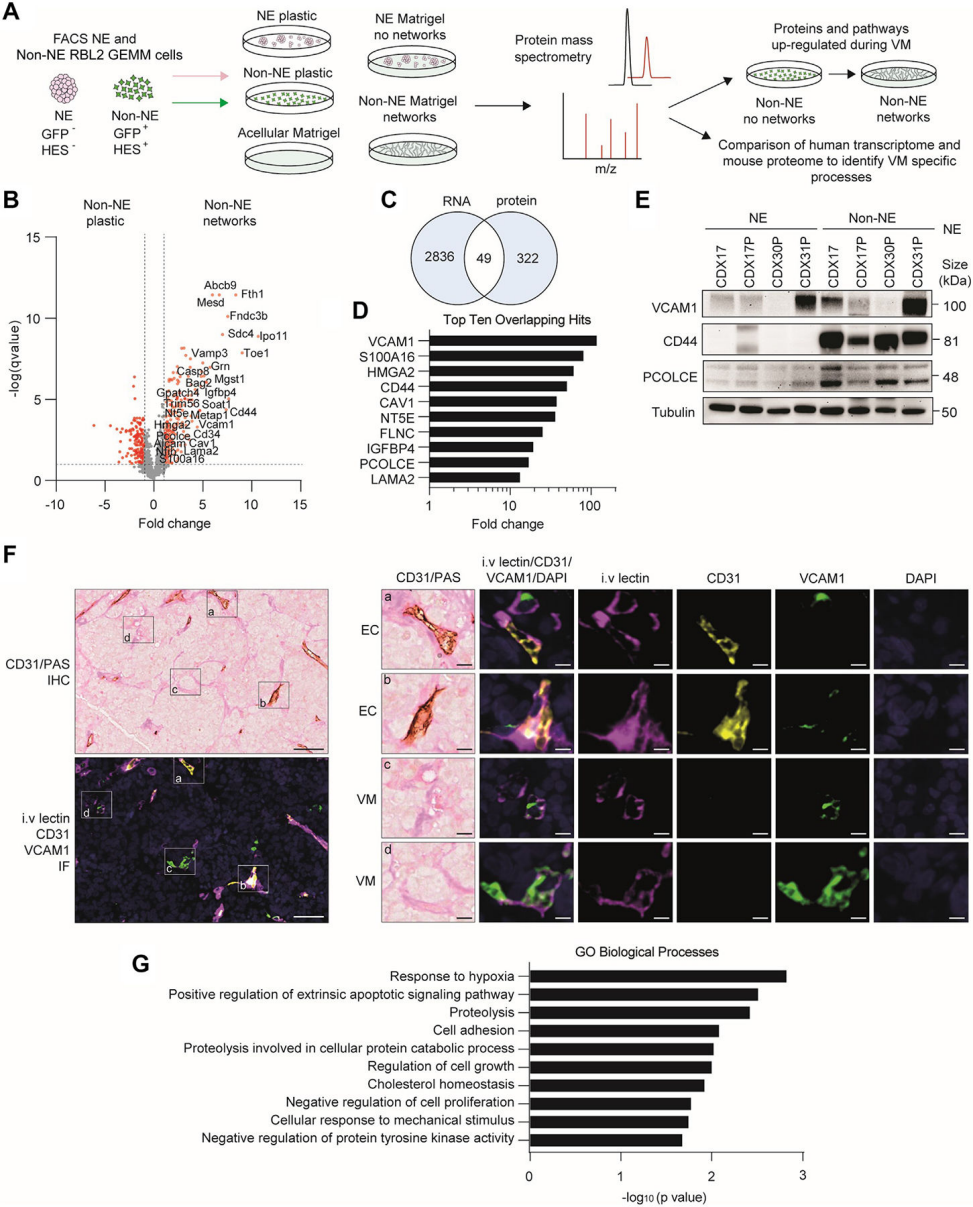
Proteomic analysis of network-forming GEMM cells. (*A*) LC-MS/MS experimental outline. NE (HES1^−^/GFP^−^) and non-NE (HES1^+^/GFP^+^) cells were generated from RBL2 GEMM tumors by flow cytometry on the basis of *Hes1*-GFP reporter expression and seeded onto plastic and Matrigel with a blank Matrigel control. Protein lysates were harvested, processed, and analyzed by LC-MS-MS in biological triplicate and technical duplicate. (*B*) Volcano plot of proteins in GEMM non-NE cells forming tubules on Matrigel versus growth on plastic. Red circles significantly differentially expressed proteins (fold change >1, −log(pvalue) >1). There were two independent tumor replicates analyzed in triplicate per condition. (*C*) Venn diagram showing 49 VM candidates overlapping between the 322 up-regulated proteins in the network-forming non-NE GEMM cells and the 2836 up-regulated genes in the CDX non-NE cells. (*D*) Transcript fold change in CDX non-NE versus NE cells of the top 10 overlapping protein and RNA hits identified in (*C*). (*E*) Representative immunoblots of CDX NE and non-NE cell lysates. There are 2 to 3 independent replicate tumors per CDX. Tubulin loading control was run subsequently for each marker illustrated on the same blot. (*F*) Representative images of CD31/PAS immunohistochemistry (top left) and intravenous tomato lectin/CD31/VCAM1/DAPI multiplex IF (bottom left) in serial tissue sections of a CDX17P tumor harvested after mice received intravenous tomato lectin injection. Individual perfused VCAM1^+^ EC vessels (images a and b; intravenous lectin^+^/CD31^+^/VCAM1^+^) and perfused VCAM1^+^ VM vessels (images c and d; intravenous lectin^+^/CD31^−^/VCAM1^+^) are illustrated on the right with single channel IF for CD31 (yellow), intravenous tomato lectin (pink), VCAM1 (green) and nuclei (DAPI, blue). Scale bars 50 *μ*m (left panels) and 10 *μ*m (right panels). (G) Gene Ontology Biological Processes representing the 49 overlapping protein and RNA hits identified in the GEMM and CDX. CDX, circulating tumor cell-derived explant; DAPI, 4’,6-diamidino-2-phenylindole; EC, endothelial cell; GEMM, genetically engineered mouse model; IF, immunofluorescence; LC-MS/MS, liquid chromatography - tandem mass spectrometry; NE, neuroendocrine; PAS, Periodic Acid Schiff stain; VM, vasculogenic mimicry.

Given the functional changes that occur during network formation without obvious alteration in gene expression in non-NE CDX cells on Matrigel versus plastic, we performed a proteomic analysis of NE and non-NE cells on both substrates to identify VM-specific changes and with acellular Matrigel incorporated as an experimental control (Fig. 5A, workflow schematic). We chose the RBL2 GEMM for this analysis to minimize patient-to-patient variability and exploit the ability to purify tumor cell subpopulations by means of fluorescence activated cell sorting of NE (GFP^−^) and non-NE (GFP^+^) cells.^11^ Differential protein expression analysis identified 332 significantly up-regulated proteins specific to network-forming non-NE cells on Matrigel (Fig. 5B; red circles fold change > 1, −log(*p* value) > 1) compared with non-NE cells on plastic or NE cells on Matrigel in which networks were not formed and in which Matrigel components were excluded.

Among the most significantly up-regulated VM-associated proteins were the cell-ECM adhesion receptor Sdc4, the endothelial cell surface receptor Cd34, cell-cell adhesion receptors Vcam1 and Cd44, and the regulator of SCLC metastasis, Nfib^57^ (Fig. 5B). We next compared these 322 up-regulated proteins with significantly up-regulated genes (*p* < 0.05, fold change > 1) in the non-NE CDX cells identified by our unbiased transcriptomics analysis on CDX models (Fig. 4I) and identified 49 overlapping VM-specific candidates (Fig. 5C). The top 10 overlapping protein and RNA hits (Fig. 5D) were up-regulated greater than 13-fold at the transcript level in non-NE compared with NE CDX cells, again identifying VCAM1 and CD44, and procollagen processing enhancer PCOLCE (Fig. 5D), which were confirmed at the protein level (Fig. 5E). Using multiplex IF in CDX17P tumors harvested from mice after intravenous lectin injection, we found that the endothelial marker VCAM1 was expressed within perfused endothelial vessels (Fig. 5F, panels a and b: intravenous lectin+/CD31+/VCAM1+) and perfused VM vessels (Fig. 5F, panels c, and d: intravenous lectin+/CD31−/VCAM1+). The intravenous lectin+/CD31−/VCAM1+ perfused VM vessels co-localized with PAS+/CD31− VM vessels in an adjacent tissue section by IHC (Fig. 5F) and combined, these data infer endothelial properties of VM vessels *in vivo*. Up-regulation of cell adhesion, cellular responses to mechanical stimuli, and hypoxia responses were revealed by GO analysis of the 49 overlapping up-regulated and VM-associated non-NE cell proteins (Fig. 5G). Overall, the combined transcriptomic and proteomic analyses signpost the importance of cell-cell and cell-ECM adhesion and ECM remodeling processes in SCLC VM (Figs. 4C and 5G).

### Integrin *β*1 Is Required For Collagen Remodeling In Vitro During Network Formation

**Figure 6.**
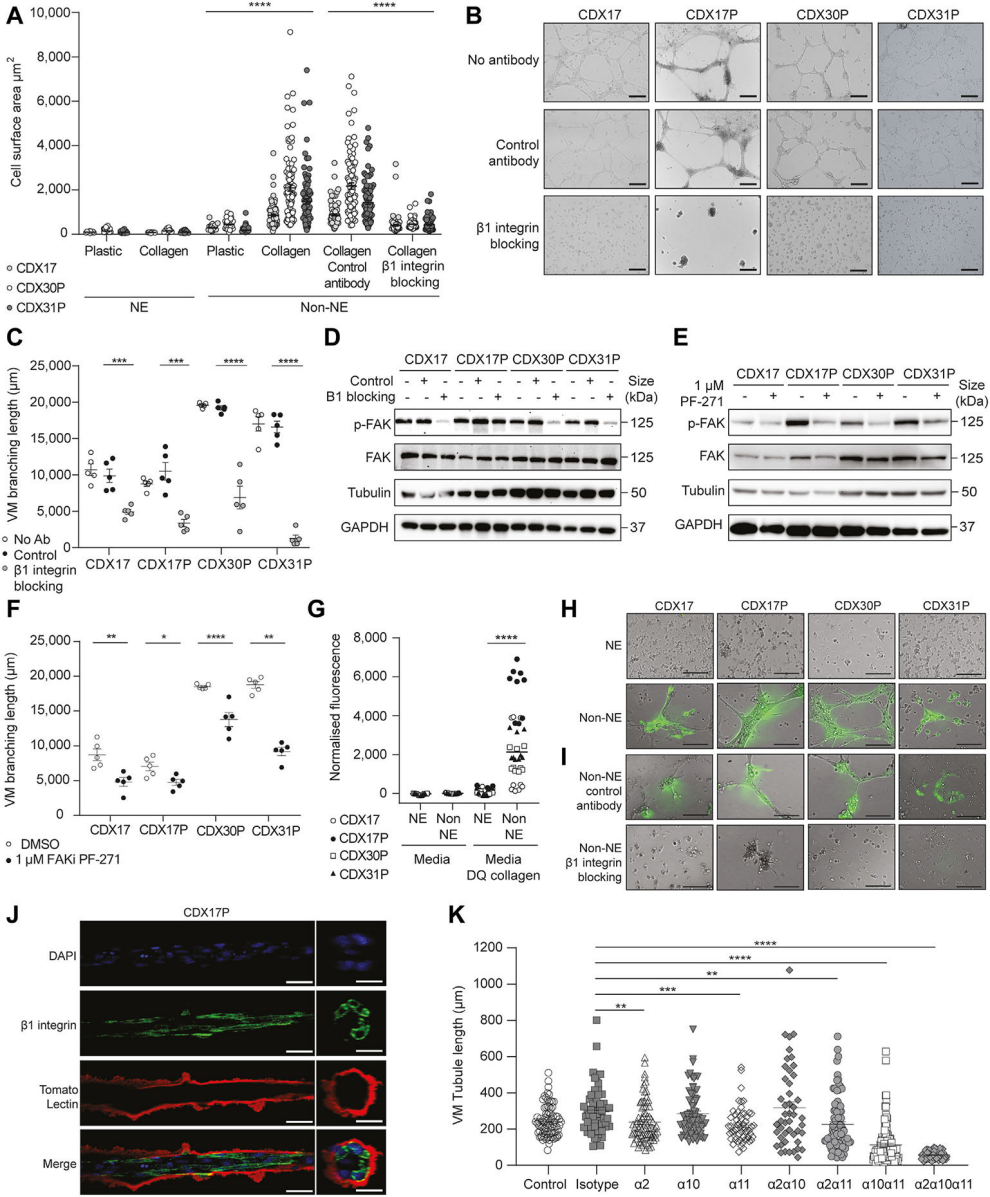
Integrin *β*1 is required for collagen remodeling in vitro during network formation. (*A*) CSA of CDX (light gray circles, CDX17, open circles, CDX30P, gray circles, CDX31P) NE and non-NE cells on plastic or collagen, and treated with an integrin *β*1 blocking antibody or isotype control antibody. Data are mean (± SEM) 200 cells analyzed from a CDX tumor (****p < 0.0001 two-tailed unpaired Student’s *t* test). (*B*) Representative images of CDX non-NE cell tubule-forming assay with no antibody, isotype control antibody, and integrin *β*1 blocking antibody. Scale bars, 500 *μ*m (n = 3 replicates per CDX tumor). (*C*) VM branching length of tubule-forming assays in (C). Data are mean (± SEM), five images analyzed per experiment (open circles, no antibody, black circles, isotype, gray circles, integrin *β*1 blocking antibody), representative of two to three replicates per CDX tumor (***p < 0.001, ****p < 0.0001 two-tailed unpaired Student’s *t* test). (*D*) Representative immunoblots of CDX non-NE cell lysates treated with no antibody, isotype control antibody, or integrin *β*1-blocking antibody (n = 3 replicates per CDX tumor). Each lysate was probed separately for p-FAK with tubulin as a loading control and total FAK with GAPDH as a loading control. (*E*) Representative immunoblots of Y397 p-FAK and FAK in CDX non-NE cell lysates treated with 1 *μ*M FAKi PF-271 or DMSO vehicle control for 24 hours, with tubulin, and GAPDH loading controls for p-FAK and FAK, respectively (n = 3 replicates per CDX tumor). (*F*) VM branching length of tubule-forming assays with PF-271 treated non-NE cells (Supplementary Fig. 6E). Data are mean (± SEM) (n = 5 images analyzed per experiment) (open circles, DMSO control; black circles, 1 *μ*M FAKi PF-271 treated cells), representative of 3 biological replicates per CDX tumor (*p < 0.05, **p < 0.005, ****p < 0.0001, two-tailed unpaired Student’s *t* test. (*G*) Fluorescence of CDX (open circles, CDX17, black circles, CDX17P, open square, CDX30P, black triangle, CDX31P) NE and non-NE cells cultured in media with or without DQ collagen. Each circle represents the fluorescence of a single well (*n* = 9 replicates per CDX tumor). Data are mean (± SEM) (****p < 0.0001 two-tailed unpaired Student’s *t* test. (*H*) Representative fluorescence images of tubule-forming assay with CDX NE and non-NE cells on Matrigel containing DQ collagen. Scale bars, 500 *μ*m (n = 2 replicates per CDX tumor). (*I*) Representative fluorescence images of CDX non-NE cell tubule-forming assay with no antibody, isotype control antibody, and integrin *β*1-blocking antibody on Matrigel containing DQ collagen. Scale bars, 500 *μ*m. (n = 2 replicates per CDX tumor). (*J*) Representative confocal microscopy images of CDX17P non-NE cells on Matrigel with immunofluorescent staining for DAPI nuclear stain (blue), membranous integrin *β*1 (green), and coated by an extracellular matrix containing lectin (tomato lectin, stained red). Images are illustrated after z-stack reconstruction using Imaris software after 72 hours on Matrigel, scale bar 50 *μ*m. (*K*) VM tubule length of CDX17P non-NE cells on Matrigel treated with antibodies blocking *α*2-, *α*10-, and *α*11- integrins, as a single agent, in dual combinations, or combined. Individual tubule length quantified using ImageJ (****p < 0.0001). Data are mean (± SEM) (*p < 0.01, **p < 0.005, ****p < 0.0001, two-tailed unpaired Student’s *t* test). CSA, cell surface area; CDX, circulating tumor cell-derived explant; DMSO, dimethylsulfoxide; DQ, dye-quenched; NE, neuroendocrine; SEM, standard error of the mean; VM, vasculogenic mimicry.

We hypothesized that the cell-ECM receptor integrin *β*1 might also be required for SCLC VM as we identified integrin signaling and ECM binding within our transcriptomics data set (Fig. 4K), and cell-ECM adhesion within our proteomics data set (Fig. 5B). Cell surface area (CSA) is a surrogate of integrin-ECM binding after short-term incubation with specific ECM substrates. CDX NE cells spread poorly both on plastic (CSA = 109 *μ*m^2^, range: 39–382 *μ*m^2^) and on collagen (CSA = 109 *μ*m^2^, range: 45–310 *μ*m^2^) whereas non-NE cells adhered partially on plastic (CSA = 304 *μ*m^2^, range: 87–972 *μ*m^2^) but exhibited significantly increased spreading on collagen (CSA = 1478 *μ*m^2^, range: 135–9122 *μ*m^2^) (*p* < 0.0001) (Fig. 6A). Non-NE cell spreading on laminin was comparable to plastic (Supplementary Fig. 6A), indicating a preferential collagen-mediated interaction. These data were recapitulated in three RBL2 GEMM cell lines derived from different mice (*p* < 0.0001) (Supplementary Fig. 6B). Cell surface expression of integrin *β* was significantly up-regulated in CDX non-NE cells compared with NE cells (*p* = 0.03) (Supplementary Fig. 6C) and perturbation of integrin-collagen interactions with an integrin *β*1–blocking antibody abrogated non-NE cell spreading on collagen (CSA = 429 *μ*m^2^ range: 137–3173 *μ*m^2^ versus CSA = 1492 *μ*m^2^, range: 234–7119 *μ*m^2^) (*p* < 0.0001) (Fig. 6A) and impaired network formation in vitro in four CDX non-NE cell cultures (Fig. 6B), in which tubule branching length was significantly reduced compared with isotype control (4150 *μ*m versus 10,461 *μ*m) (*p* < 0.0001) (Fig. 6C). Cytoskeletal changes transduced by means of FAK activation downstream of integrin *β*1–ECM binding^58^ were also implicated in VM network formation as integrin *β*1 blockade reduced FAK phosphorylation (p-FAK) in 3 of 4 CDX non-NE cell cultures (Fig. 6D). Pharmacologic inhibition of FAK with the potent adenosine triphosphate–competitive reversible FAK inhibitor PF-5 65,271^59^ reduced levels of p-FAK at non-toxic conentrations (Fig. 6E and Supplementary Fig. 6D) and impaired in vitro VM network formation in four CDX non-NE cell cultures (Supplementary Fig. 6E), with a significant reduction in VM branching length compared with the DMSO control (Fig. 6F) (CDX17, *p* = 0.006; CDX17P, *p* = 0.02; CDX30P, *p* < 0.0001; CDX31P, *p* = 0.002). Collectively, these data indicate that integrin *β*1–ECM engagement and intracellular integrin signaling by means of FAK is important for CDX non-NE cell VM network formation in vitro.

Because non-NE cells require integrin *β*1 to adhere to collagen-rich ECM in Matrigel and in microvascular basement membranes^60^ we hypothesized that non-NE cells remodel collagen into VM structures both on Matrigel and in CDX tumors, identified by PAS-lined VM vessels (Fig. 1). As a surrogate for collagen remodeling *in vivo*, we measured collagen cleavage by culturing CDX non-NE and NE cells in the presence of DQ collagen which fluoresces when cleaved. Non-NE cells cleaved significantly more (45-fold, *p* < 0.0001) DQ collagen than NE cells (Fig. 6G) and fluorescent non-NE cell tubules incorporated DQ collagen (Fig. 6H). Inhibition of tubule formation by means of integrin *β*1 blockade diminished DQ collagen incorporation into non-NE cells suggesting collagen remodeling is required for network formation (Fig. 6I). PSR staining visualizes collagen fiber organization in cells and tissues and confirms that collagen was remodeled by non-NE CDX cells forming networks on Matrigel (Supplementary Fig. 6F). PAS glycoprotein stain that detects basement membranes and VM vessels in tissues was also confined to regions containing non-NE cells within networks (Supplementary Fig. 6G and H). Given that we found that hollow tubule formation by CDX non-NE cells on Matrigel (Fig. 3E and F) occurs by means of integrin *β*1–mediated remodeling of ECM glycoproteins (Fig. 6I and Supplementary Fig. 6F), we visualized tubule-forming CDX non-NE cells which had been fluorescently labeled with DAPI nuclear stain (blue), integrin *β*1 (green) and tomato lectin (red), the latter of which recognizes poly-*N*-acetyl lactosamine-type oligosaccharide moieties present in ECM^61^ (Fig. 6J). The IF revealed that integrin *β*1–expressing CDX cells formed multicellular hollow tubules that interact with an outer glycoprotein-rich Matrigel shell labeled by lectin that is not present within the tubule lumen (Fig. 6J), further supporting a functional role of integrin *β*1 in hollow tubule formation by SCLC non-NE cells. Because integrin *β*1 is required for collagen binding by means of heterodimerization with integrin *α* subunits (*α*1, *α*2, *α*3, *α*10, and *α*11) and the CDX non-NE cells up-regulate integrin *α*2, integrin *α*10, and integrin *α*11 at the cell surface (Supplementary Fig. 6I), we sought to explore the importance and functional redundancy of collagen specific integrin *β*1–binding partners in VM. We inhibited integrin *α*2, integrin *α*10, and integrin *α*11 with function-blocking antibodies in CDX17P non-NE cells, as single agents, and in combinations, then assessed VM network formation on Matrigel. The inhibition of integrin *α*2 and integrin *α*11 alone significantly reduced VM network formation on Matrigel (Fig. 6K) (integrin *α*2, *p* = 0.0019; integrin *α*11, *p* = 0.0002) and dual combinations with integrin *α*11 further reduced VM network formation (Fig. 6K) (integrins *α*2*α*11, *p* = 0.0013; integrins *α*10*α*11, *p* < 0.0001). Inhibition of all three-collagen binding integrin *α* subunits completely abolished VM network formation (Fig. 6I) (*p* < 0.0001), comparable to inhibition of integrin *β*1 (Fig. 6C), further substantiating our findings that collagen binding is essential for VM network formation. Overall, these findings indicate that SCLC non-NE cells require integrin *β*1 to interact with collagen in the ECM, which is remodeled into hollow branching networks.

## Discussion

Robust, patient-representative preclinical models of SCLC support the characterization of intra- and intertumoral heterogeneity.^8,9,14,23,62^ VM is emerging as a phenomenon exploited by aggressive cancers to maintain their supply of oxygen and nutrients to support growth and metastasis.^17,18^ Having previously reported that VM correlates with poor prognosis in patients with SCLC,^21^ we sought to identify VM cellular machinery and explore underlying molecular mechanisms using our CDX biobank, which has a range of VM profiles (Fig. 1). We exhibited here that non-NE cell subpopulations are responsible for VM in human CDX and GEMM SCLC tumors (Figs. 1-3), and that VM correlates with tumor growth without increased angiogenesis (Fig. 1).

Cancer cells undergo lineage plasticity to adapt, survive therapy, grow, and metastasize.^63^ NOTCH-induced transition of NE to non-NE cells occurred in *ex vivo* cultures of an ASCL1 subtype CDX, which was required for VM (Fig. 3), validating the SCLC plasticity seen in GEMMs.^9-11^ Non-NE CDX cells formed hollow tubules on Matrigel with a lumen diameter supportive of erythrocyte transit that closely resembled structures formed by HUVECs under identical conditions (Fig. 3). Combined with *in vivo* data showing that lectin-perfused, VCAM1-positive VM vessels lacking CD31-positive murine endothelial cells are present in CDX tumors *in vivo* (Figs. 1 and 5), this provides evidence that human tumor cells form VM vessels with endothelial properties enabling them to act as functional blood vessels *in vivo*.

*Ex vivo* cultures of non-NE cells from four CDX models exhibited enhanced attachment to plastic, downregulated NE markers, and up-regulated non-NE markers including REST and YAP1,^41^ relative to their suspension NE counterparts (Fig. 3). Non-NE cells expressed at least one NOTCH receptor (NOTCH1 or NOTCH2, or both) and increased MYC (Fig. 3), and NOTCH activation induced MYC expression (Fig. 3). With recent findings that MYC and NOTCH co-operate to drive NE to non-NE transition in SCLC RPM GEMM tumors,^9^ these additional data in CDX models suggests that MYC signaling may be another important driver of NE to non-NE plasticity in SCLC to enable VM. Co-operation between non-NE and NE cells is essential for metastasis in the RP (*Trp53*^*fl/fl*^/*Rb1*^*fl/fl*^) SCLC GEMM^13^ and VM is required for metastasis in a breast cancer model.^17^ Studies to evaluate relationships between VM, MYC, NOTCH, and metastasis in SCLC CDX are underway.

A surprising result of this study was the broadly similar transcriptomes of non-NE cells on plastic (monolayers) and Matrigel (networks), suggesting that phenotypic switching from NE cells was accompanied by the acquisition of a transcriptional program, poising cells for VM in a conducive microenvironment (Fig. 4). Non-NE cells up-regulated the hypoxia-responsive TF *FOXC2* (Fig. 4H), a key driver of VM and resistance to anti-angiogenic therapy in aggressive tumor subtypes.^46^ Hypoxia promotes VM in melanoma, colorectal cancer, and Ewing sarcoma,^20,50,51^ and although SCLC is fast-growing and typically hypoxic,^64^ network formation by non-NE cells occurred in oxygenated Matrigel. However, normoxic non-NE cells expressed hypoxia gene signatures, stabilized HIF1*α*, and up-regulated downstream effectors (Fig. 4) and the hypoxia response was also identified as the top GO biological process when analyzing the 49 overlapping proteomic and transcriptomic hits identified in the GEMM and CDX (Fig. 5). Therefore, unlike other cancer types, physiological hypoxia may not be essential for SCLC VM in non-NE cells because they are pseudohypoxic, whereby the non-NE cells have retained features of previous physiological hypoxia *in vivo*. Given the roles of NOTCH and MYC in SCLC NE to non-NE plasticity, it is notable that NOTCH, MYC, and hypoxia signaling are also entwined in regulating endothelial angiogenesis,^65,66^ suggesting that they are key factors in tumor-endothelial differentiation in SCLC. Moreover, HIF-1*α* can bind to the NOTCH intracellular domain to augment NOTCH signaling,^67^ suggestive of a forward signaling loop once non-NE cells acquire pseudohypoxic traits. HIF1-*α* interacts and co-operates with oncogenic MYC to enhance expression of target genes including those involved in metabolic adaptation and the Warburg effect. Further studies are now warranted to understand the intricacies and importance of pseudohypoxia in MYC and NOTCH-expressing non-NE cells undergoing VM.

We found that non-NE CDX cells remodeled ECM components in Matrigel to form hollow tubular networks lined by ECM and co-localized with integrin *β*1–expressing cells (Fig. 6). Integrin *β*1 is widely expressed,^68^ present in both NE and non-NE SCLC cells. Integrin *β*1 expression is associated with poorer prognosis in patients with SCLC^69^ and metastasis in an SCLC allograft model by means of FAK signaling.^70^ The role(s) of integrin *β*1 in NE and non-NE SCLC cells are unexplored. Here, we found that only non-NE cells are primed with active integrin *β*1 to enable VM network formation on Matrigel with the requirement of downstream FAK activation (Fig. 6). FAK activation is also a requirement for metastatic melanoma VM.^18^ The remodeling of collagen in Matrigel by non-NE cells (Fig. 6) was consistent with up-regulation of PCOLCE (Figs. 4 and 5), a protein that binds to and enhances the activity of collagen C-proteinase to enable fibrillar collagen formation.^71^ Collagen architecture regulates cancer cell motility, invasiveness, and metastasis; collagen-rich tumor regions, akin to VM vessel-dense regions identified in CDX (Fig. 1), are associated with aggressive cell phenotypes.^72^ Furthermore, VM-forming breast cancer cells are exquisitely sensitive to changes in collagen or- ganization,^42^ further supporting the importance of integrin-mediated ECM remodeling in VM. The CDX non- NE cells required collagen-binding integrin *a* subunits to form VM networks on Matrigel (Fig. 6). The *ITGA11* gene was the most highly up-regulated integrin *α* subunit in the CDX non-NE cells and inhibition of integrin *α*11, alone or in combinations with other collagen-binding integrin *α* subunits, consistently inhibited VM. Whereas integrin *α*11 has not been previously identified in VM, its expression in NSCLC cancer-associated fibroblasts fuels tumor growth and metastasis,^73^ supporting its possible role in SCLC VM and tumor progression. Further experiments aim to identify the full complement of essential integrin *β*1 *α* binding partners required for SCLC VM and explore their role as tractable drug targets.

In summary, we report a new aspect of SCLC intratumoral functional heterogeneity that arises from lineage plasticity when NE cells, the major cell type in most SCLC tumors, transition to a non-NE phenotype that can mimic endothelial cell behaviors by means of VM. Together with findings that non-NE cells are more chemoresistant and required for SCLC metastasis,^11,13,41^ this study strongly recommends that therapeutic strategies should broaden beyond targeting NE biology and co-target VM-competent non-NE cells.

## Supplementary Material

Supplementary Tables

Supplementary Data

Figure S3

Figure S4

Figure S5

Figure S6

Figure S1

Figure S2
