# Supplementary material for: Lineage Plasticity in SCLC Generates Non-Neuroendocrine Cells Primed for Vasculogenic Mimicry
Source: J Thorac Oncol. Author manuscript; Available in PMC 2023 Oct 9. (PMC10561473; doi:10.1016/j.jtho.2023.07.012)
Supplement: Supplementary Tables [file NIHMS1933668-supplement-Supplementary_Tables.docx]

**Supplementary Table 1, Related to Figure 1 and Supplementary Figure 1. SCLC subtype classification (ASCL1, NEUROD1, POU2F3 and ATOH1) ^8^ and YAP1 RNA and/or protein expression ^27^ of the CDX models used in this study.**

| **CDX** | **Subtype (Simpson et al., 2020)^8^** | **YAP1 (Pearsall et al., 2020)^27^** |
| --- | --- | --- |
| CDX01 | ASCL1 | RNA |
| CDX02 | ASCL1 |  |
| CDX03 | ASCL1 |  |
| CDX03P | ASCL1 |  |
| CDX08 | NEUROD1 |  |
| CDX08P | NEUROD1 |  |
| CDX09 | ASCL1 |  |
| CDX10 | ASCL1 |  |
| CDX12 | ASCL1 | Protein |
| CDX13 | POU2F3 |  |
| CDX14P | ASCL1 |  |
| CDX15P | ASCL1 | RNA and protein |
| CDX15PP | ASCL1 | RNA and protein |
| CDX17 | ATOH1 |  |
| CDX17P | ATOH1 |  |
| CDX18 | ASCL1 | RNA |
| CDX18P | ASCL1 | RNA |
| CDX20 | ASCL1 |  |
| CDX20P | ASCL1 |  |
| CDX21* | NEUROD1 | Protein |
| CDX22P | ASCL1 | RNA and protein |
| CDX25 | ATOH1 | RNA |
| CDX29 | NEUROD1 |  |
| CDX30P | ATOH1 | RNA and protein |
| CDX31P | ASCL1 | RNA and protein |

P refers to a model made from a blood sample taken during post therapy disease progression. PP refers to model derived from a second post chemotherapy longitudinal blood sample. * denotes CDX21, a newly characterized model used and phenotyped in this study (Figure S1).

**Supplementary Table 2, Related to Figure 1. Clinical Characteristics of the 20 SCLC patients whose tumor biopsies were stained for VM.**

| **CDX No.** | **Patient No.** | **Gender** | **Age at Diagnosis (years)** | **SCLC Stage** | **Site(s) of Metastases** | **1st Line Treatment** | **Treatment Response** | **Overall Survival (months)** |
| --- | --- | --- | --- | --- | --- | --- | --- | --- |
| N/A | 83 | Male | 82 | Extensive | Lung, Pleural Effusion, Liver, Adrenal, Nodes | Carboplatin/Etoposide | Stable Response | 4.2 |
| N/A | 218 | Male | 56 | Limited | N/A | Carboplatin/Etoposide | Partial Response | Not Reached  167 |
| N/A | 220 | Female | 50 | Extensive | Bone | Carboplatin/Etoposide | Progressive Disease | 12.7 |
| N/A | 235 | Female | 74 | Limited | N/A | Carboplatin/Etoposide | Partial Response | 11.2 |
| N/A | 270 | Female | 66 | Limited | N/A | Carboplatin/Etoposide | Unassessed | 2.7 |
| N/A | 309 | Male | 50 | Limited | N/A | Cisplatin/Etoposide | Partial Response | 17.8 |
| N/A | 320 | Male | 72 | Extensive | Nodes, Liver, Bone | Carboplatin/Etoposide | Unassessed | 2.5 |
| N/A | 365 | Female | 69 | Extensive | Pleural Effusion, Pancreas, Adrenal, Spleen | Carboplatin/Etoposide | Partial Response | 5.2 |
| N/A | 390 | Female | 81 | Extensive | Nodes, Liver | Carboplatin/Etoposide | Unassessed | 3.0 |
| N/A | 432 | Female | 74 | Extensive | N/A | Carboplatin/Etoposide | Unassessed | 4.9 |
| N/A | 12013 | Female | 48 | Extensive | Nodes, Lung, Pleural Effusion, Adrenal, Bone | Carboplatin/Etoposide | Progressive Disease | 13.1 |
| N/A | 12023 | Female | 64 | Extensive | Pleura | Carboplatin/Etoposide |  | 1.7 |
| N/A | 12077 | Female | 63 | Extensive | Nodes, Pleura, Liver | VAC | Partial Response | 8.2 |
| 12 | 12106 | Male | 74 | Extensive | Liver, Pleural Effusion | Carboplatin/Etoposide | Partial Response | 5.2 |
| N/A | 12114 | Female | 63 | Extensive | Bone, Liver | Carboplatin/Etoposide | Partial Response | 8.0 |
| N/A | 12151 | Female | 50 | Extensive | Nodes, Bone, Liver | Carboplatin/Etoposide | Partial Response | 18.2 |
| N/A | 12152 | Male | 73 | Extensive | Liver, Adrenal | Carboplatin/Etoposide |  | 3.5 |
| 25 | 12265 | Male | 80 | Extensive | Nodes, Liver | Carboplatin | Progressive disease | 2.4 |
| N/A | 12278 | Male | 64 | Extensive | Lung, Nodes | Carboplatin/Etoposide | Partial Response | 8.4 |
| N/A | 12370 | Female | 48 | Extensive | Axilla Node | Carboplatin/Etoposide | Progressive disease | 10.6 |

**Supplementary Table 3, Related to Figure 4. NOTCH pathway and MYC family member expression in CDX NE versus non-NE cells.**

| **Gene** | **Fold change NE vs non-NE** | **Adjusted p-value** |
| --- | --- | --- |
| **Upregulated in non-NE** |  |  |
| NOTCH2 | 5.4 | 7.4E-26 |
| NOTCH3 | 12.8 | 3.1E-36 |
| HES1 | 2.2 | 2.5E-06 |
| MYC | 9.3 | 4.5E-19 |
| **Upregulated in NE** |  |  |
| DLL1 | 22.0 | 6.2 E-18 |
| DLL3 | 4.2 | 1.6E-12 |
| DLL4 | 13.0 | 9.1E-14 |
| MYCL | 14.2 | 2.1E-07 |

Notch pathway receptors (*NOTCH2* and *NOTCH3*), NOTCH pathway ligands (*DLL1, DLL3* and *DLL4*), NOTCH effector (*HES1*) and MYC family member (*MYC, MYCL)* transcript fold change and adjusted p-values in CDX NE versus non-NE cells.

**Supplementary Table 4.xml, Related to Figure 4. Endothelial specific, blood vessel development, angiogenesis and coagulation that are significantly up-regulated in CDX non-NE cells**

**Supplementary Table 5. Antibodies used for immunohistochemistry.** Ready to use (RTU)

| Antibody | Company | Dilution | Antigen Retrieval | Incubation |
| --- | --- | --- | --- | --- |
| REST | ThermoFisher /MA5-24606 | 1:150 | pH6 20’ | 60’ |
| Synaptophysin | Leica Biosystems/pA0299 | RTU | pH9 20’ | 20’ |
| CD31 (mouse) | Abcam/ab124432 | 1:400 | pH 6 20’ | 20’ |
| CD31 (IF) | Cell Signaling/77699 | 1:200 | pH 6’20’ | 30’ |
| CD31 (human) | DAKO/M0823 | 1:40 | pH 6’20’ | 20’ |
| ASCL1 | BD Pharminigen/556604 | 1:250 | pH6 20’ | 20’ |
| NEUROD1 | Abcam/ab213725 | 1:100 | pH6 10’ | 20’ |
| YAP1 | Abcam/ab52771 | 1:100 | pH6 20’ | 20’ |
| POU2F3 | Sigma-Aldrich/HPA0196652 | 1:250 | pH6 20’ | 20’ |
| NCAM1 | Leica Biosystems/CD56-504-L-CE | 1:100 | pH6 20’ | 20’ |
| TTF1 | DAKO/M3575 | 1:200 | pH9 40’ | 16’ |
| cMYC | Abcam/ab32072 | 1:75 | pH6 20’ | 30’ |
| Cytokeratin | Dako/M3515 | 1:100 | pH6 20’ | 20’ |
| Vimentin | Ventana/790 2917 | RTU | pH9 32’ | 16’ |
| VCAM1 | Abcam/ ab134047 | 1:1000 | pH6 20’ | 30’ |
| EpCAM | Cell Signaling/2929 | 1:100 | pH6 30’ | 20’ |

**Supplementary Table 6. Antibodies used for immunoblotting**

| Antibody | Company | Clone | Dilution |
| --- | --- | --- | --- |
| ASCL1 | BD Pharminigen/556604 | 24B72D11.1 | 1:500 |
| NEUROD1 | Abcam/ab213725 | EPR20766 | 1:500 |
| Synaptophysin (SYP) | Abcam/ab32127 | YE269 | 1:20000 |
| REST | LifeSpan Biosciences/LS-C668231 | N/A | 1:500 |
| YAP1 | Abcam/ab52771 | EP1674Y | 1:1000 |
| NOTCH1 | Bethyl laboratories/A301-895A | N/A | 1:500 |
| NOTCH2 | Bethyl laboratories/A302-083A | N/A | 1:500 |
| cMYC | Abcam/ab32072 | Y69 | 1:500 |
| Vimentin | Cell Signaling Technologies/CST5741 | D21H3 | 1:500 |
| HIF-1a | Abcam/ab51608 | EP1215Y | 1:500 |
| CA9 | Novus Biologicals/NB100-417 | N/A | 1:500 |
| GLUT1 | Abcam/ab115730 | EPR3915 | 1:10000 |
| COL1A1 | Abcam/ab34710 | N/A | 1:1000 |
| ITGA11 | R&D Systems/AF4235 | N/A | 1:500 |
| AXL | Cell Signalling Technologies/CST8661 | C89E7 | 1:500 |
| VCAM1 | R&D Systems/BBA5 | BBIG-V1 | 1:500 |
| PCOLCE | R&D Systems/MAB2627 | 261730 | 1:500 |
| CD44 | Abcam/ab157107 | N/A | 1:1000 |
| YAP1 | Abcam/ab52771 | EP1674Y | 1:500 |
| Tubulin | Cell Signaling Technologies/CST2144 | N/A | 1:1000 |
| GAPDH | Cell Signaling Technologies/CST2118 | 14C10 | 1:1000 |
| FAK | BD Biosciences, 610088 | 77/FAK | 1:150 |
| Phospho-FAK (Tyr397) | ThermoFisher Scientific, 44624G | N/A | 1:500 |
