# Supplementary material for: Lineage Plasticity in SCLC Generates Non-Neuroendocrine Cells Primed for Vasculogenic Mimicry
Source: J Thorac Oncol. Author manuscript; Available in PMC 2023 Oct 9. (PMC10561473; doi:10.1016/j.jtho.2023.07.012)
Supplement: Figure S4 [file NIHMS1933668-supplement-Figure_S4.pptx]

## Slide 1
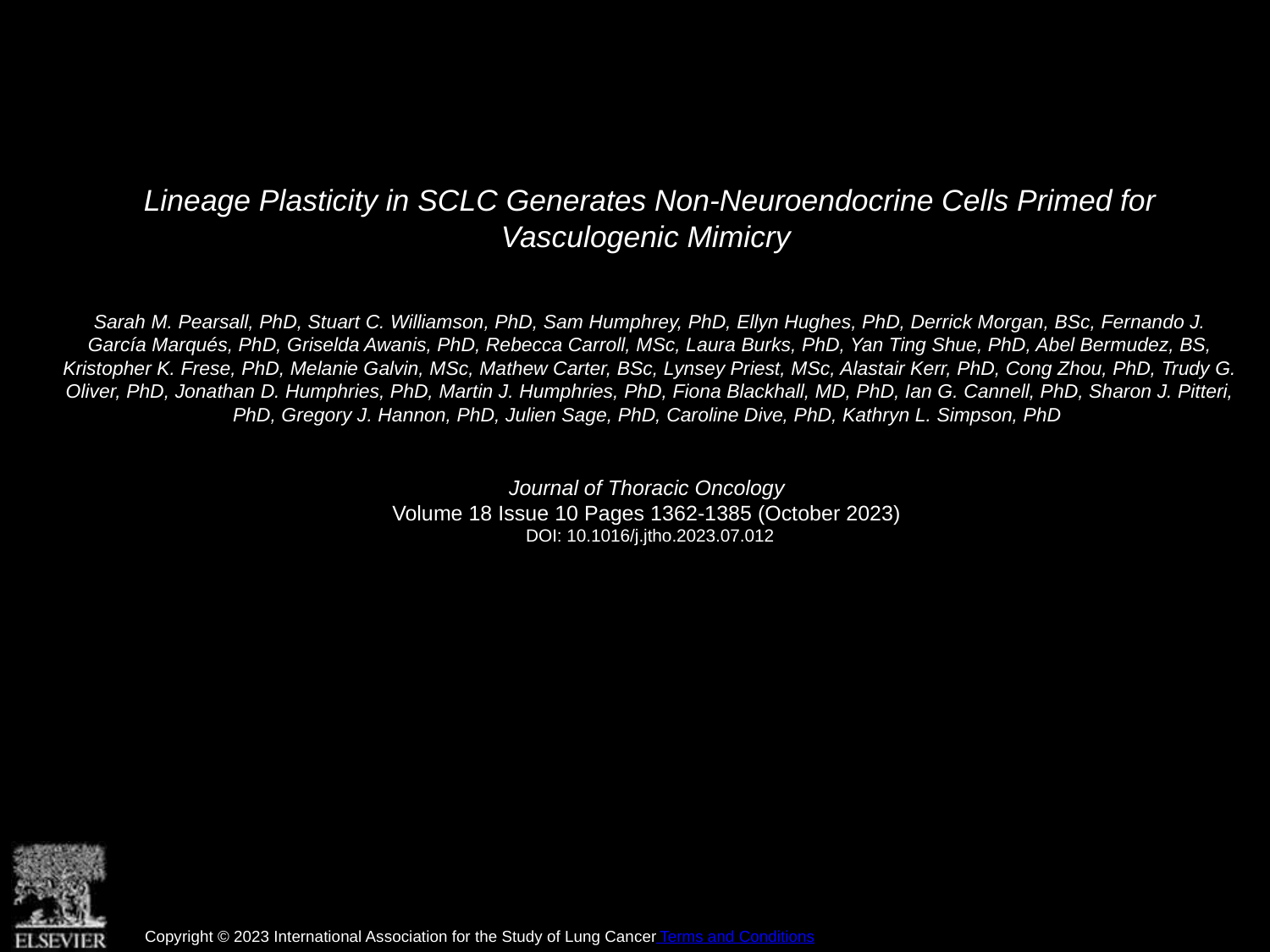

Lineage Plasticity in SCLC Generates Non-Neuroendocrine Cells Primed for Vasculogenic Mimicry
Sarah M. Pearsall, PhD, Stuart C. Williamson, PhD, Sam Humphrey, PhD, Ellyn Hughes, PhD, Derrick Morgan, BSc, Fernando J. García Marqués, PhD, Griselda Awanis, PhD, Rebecca Carroll, MSc, Laura Burks, PhD, Yan Ting Shue, PhD, Abel Bermudez, BS, Kristopher K. Frese, PhD, Melanie Galvin, MSc, Mathew Carter, BSc, Lynsey Priest, MSc, Alastair Kerr, PhD, Cong Zhou, PhD, Trudy G. Oliver, PhD, Jonathan D. Humphries, PhD, Martin J. Humphries, PhD, Fiona Blackhall, MD, PhD, Ian G. Cannell, PhD, Sharon J. Pitteri, PhD, Gregory J. Hannon, PhD, Julien Sage, PhD, Caroline Dive, PhD, Kathryn L. Simpson, PhD
Journal of Thoracic Oncology
Volume 18 Issue 10 Pages 1362-1385 (October 2023)
DOI: 10.1016/j.jtho.2023.07.012
Copyright © 2023 International Association for the Study of Lung Cancer Terms and Conditions

## Slide 2
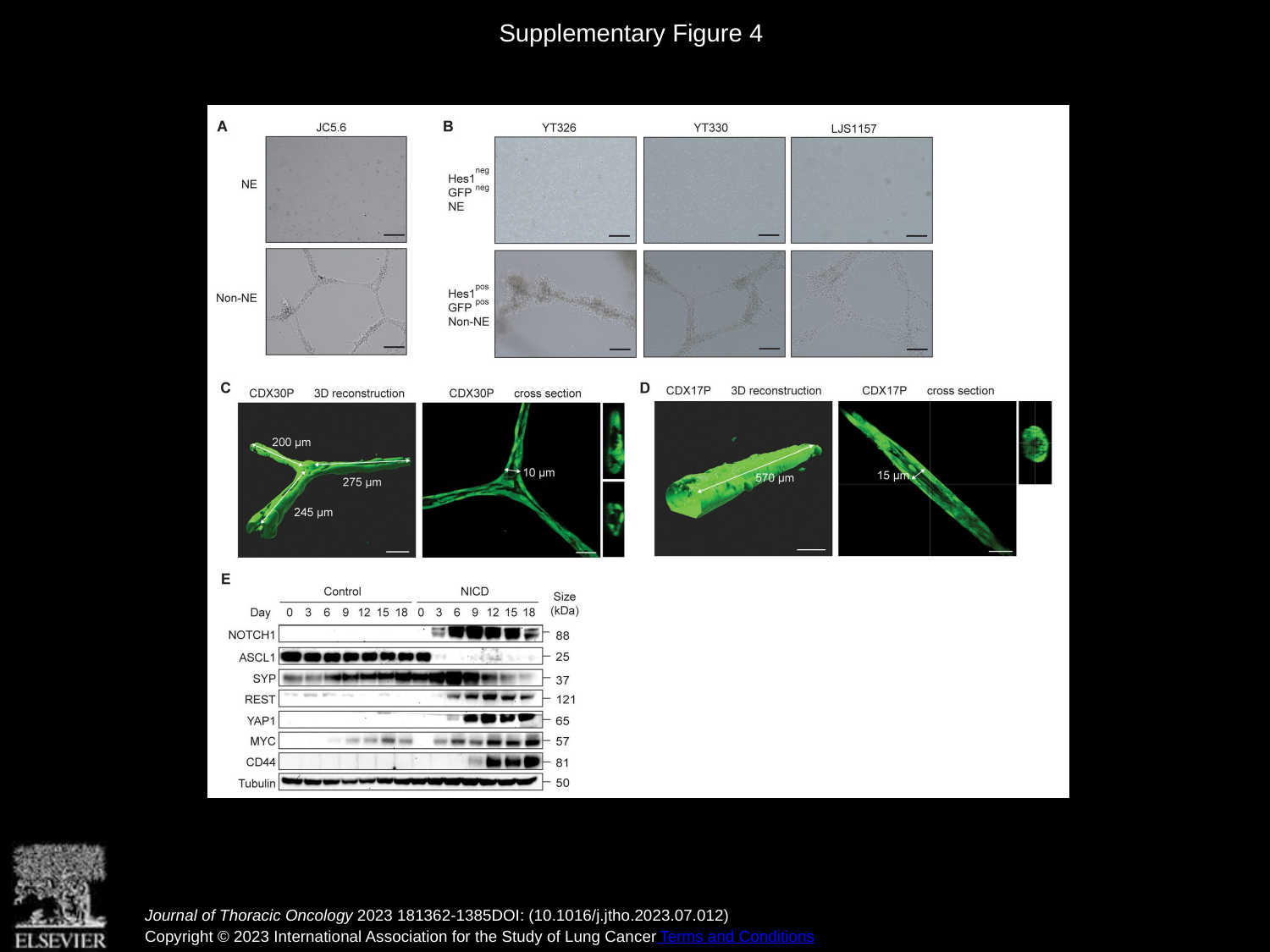

Supplementary Figure 4
Journal of Thoracic Oncology 2023 181362-1385DOI: (10.1016/j.jtho.2023.07.012)
Copyright © 2023 International Association for the Study of Lung Cancer Terms and Conditions
